# Immunohistochemical, pharmacovigilance, and omics analyses reveal the involvement of ATP-sensitive K^+^ channel subunits in cancers: role in drug–disease interactions

**DOI:** 10.3389/fphar.2023.1115543

**Published:** 2023-04-25

**Authors:** Fatima Maqoud, Nicola Zizzo, Marcella Attimonelli, Antonella Tinelli, Giuseppe Passantino, Marina Antonacci, Girolamo Ranieri, Domenico Tricarico

**Affiliations:** ^1^ Functional Gastrointestinal Disorders Research Group, National Institute of Gastroenterology Saverio de Bellis, I.R.C.C.S. Research Hospital, Milan, Italy; ^2^ Section of Pharmacology, Department of Pharmacy-Pharmaceutical Sciences, University of Bari “Aldo Moro”, Bari, Italy; ^3^ Section of Veterinary Pathology and Comparative Oncology, Department of Veterinary Medicine, University of Bari “Aldo Moro”, Valenzano, Italy; ^4^ Department of Biosciences, Biotechnologies, and Biopharmaceutics, University “Aldo Moro” Bari, Bari, Italy; ^5^ Department of Interventional Radiology and Integrated Medical Oncology, I.R.C.C.S. Istituto Tumori “Giovanni Paolo II”, Bari, Italy

**Keywords:** immunohistochemistry, cancer, Cantu’ syndrome, KATP channel genes, omics analysis, pharmacovigilance analysis

## Abstract

**Background:** ATP-sensitive-K+ channels (KATP) are involved in diseases, but their role in cancer is poorly described. Pituitary macroadenoma has been observed in Cantu’ syndrome (C.S.), which is associated with the gain-of-function mutations of the *ABCC9* and *KCNJ8* genes. We tested the role of the *ABCC8*/Sur1, *ABCC9*/Sur2A/B, *KCNJ11*/Kir6.2, and *KCNJ8*/Kir6.1 genes experimentally in a minoxidil-induced renal tumor in male rats and in the female canine breast cancer, a spontaneous animal model of disease, and in the pharmacovigilance and omics databases.

**Methods:** We performed biopsies from renal tissues of male rats (*N* = 5) following a sub-chronic high dosing topical administration of minoxidil (0.777–77.7 mg/kg/day) and from breast tissues of female dogs for diagnosis (*N* = 23) that were analyzed by immunohistochemistry. Pharmacovigilance and omics data were extracted from EudraVigilance and omics databases, respectively.

**Results:** An elevated immunohistochemical reactivity to Sur2A-mAb was detected in the cytosol of the Ki67+/G3 cells other than in the surface membrane in the minoxidil-induced renal tumor and the breast tumor samples. *KCNJ11, KCNJ8*, and *ABCC9* genes are upregulated in cancers but *ABCC8* is downregulated. The Kir6.2-Sur2A/B-channel opener minoxidil showed 23 case reports of breast cancer and one case of ovarian cancer in line with omics data reporting, respectively, and the negative and positive prognostic roles of the *ABCC9* gene in these cancers. Sulfonylureas and glinides blocking the pancreatic Kir6.2-Sur1 subunits showed a higher risk for pancreatic cancer in line with the positive prognostic role of the *ABCC8* gene but low risks for common cancers. Glibenclamide, repaglinide, and glimepiride show a lower cancer risk within the KATP channel blockers. The Kir6.2-Sur1 opener diazoxide shows no cancer reactions.

**Conclusion:** An elevated expression of the Sur2A subunit was found in proliferating cells in two animal models of cancer. Immunohistochemistry/omics/pharmacovigilance data reveal the role of the Kir6.1/2-Sur2A/B subunits as a drug target in breast/renal cancers and in C.S.

## Introduction

The ATP-sensitive potassium (KATP) channels are complexes of inwardly rectifier K^+^ subunits Kir6.1 and Kir6.2 associated with regulatory sulfonylureas receptor (Sur1 and Sur2 and its splicing products Sur2A and 2B) subunits. The Kir6.1-Sur2B, Kir6.2-Sur2A, and Kir6.2-Sur1 are the main complexes in cardiovascular, skeletal muscles, and pancreatic beta cells, respectively (Ashcroft, 2006; Flagg et al., 2010). KATP channels are stimulated by low intracellular ATP/ADP ratio, second messengers (Hons and Singh, 2005; Mele et al., 2012), kinases like AMPK (López-Gambero et al., 2021), and hormones including insulin (Tricarico et al., 1997; 2003b). KATP channels are involved in diabetes mellitus and hyperinsulinemia, hypertension, angina, cardiac ventricular dysfunction and arrhythmias, ischemia–reperfusion (Ashcroft, 2006; Olson and Terzic, 2010), neurodegeneration (Maqoud et al., 2022) and addiction (López-Gambero et al., 2021), pain and migraine (Fisher et al., 2019; Haanes and Edvinsson, 2019; Luu et al., 2019), and in the KATP channelopathies (Olson and Terzic, 2010; Nichols et al., 2013; Scala et al., 2020; 2021).

The KATP channels are relevant targets for drugs of therapeutic interest in type II diabetes , congenital neonatal diabetes (Tricarico et al., 2000; 2003a; Ashcroft, 2006), alopecia (Tricarico et al., 2018), hypertension, angina (Olson and Terzic, 2010), Cantu’ syndrome (Nichols et al., 2013), and in neurodegenerative-inflammatory disorders (Maqoud et al., 2022) and target for cytotoxic drugs (Mele et al., 2014). Estrogens may differently regulate Kir6.2 and Sur2A-B in some tissues, for instance, upregulating them in cardiomyocytes and downregulating KATP channel subunits in neurons from female rats with opposite effects (Brown et al., 2005; Niu et al., 2011) with gender differences.

Ion channels are well-known factors regulating cell proliferation through several mechanisms interfering with the cell cycle and mitosis (Dinardo et al., 2012; Tricarico et al., 2013; Becchetti et al., 2019; Rosendo-Pineda et al., 2020). *In vitro* and *ex vivo* investigations evidenced that the KATP channel subunits are functionally expressed in multiple types of cancer cells, including hepatocellular carcinoma (Malhi et al., 2000), human bladder cancer (Monen et al., 1998), human gastric cancer, and glioma (Huang et al., 2009). KATP channel openers (minoxidil, cromakalim, and pinacidil) increase the proliferation of HepG2 liver cancer cells, whereas KATP channel blockers (quinidine and glibenclamide) reduce cell proliferation (Malhi et al., 2000). Glibenclamide inhibits proliferation and induces apoptosis in bladder cell carcinoma, prostate cancer, and gastric cancer (Qian et al., 2008). Glibenclamide also inhibits cell growth by inducing G0/G1 arrest in the human breast cancer cell line MDA-MB-231 (Núñez et al., 2013). Increased KATP channel expression and activity were observed in high-grade, poorly differentiated, and invasive human cervical cancer biopsies (VázquezSánchez et al., 2018), and glibenclamide reverted cell proliferation in these cells. Glibenclamide inhibits multidrug resistance protein (MRP1) activity in human lung cancer cells (Payen et al., 2001), and more interestingly, KATP channel openers have a role in increasing the permeability of the blood–brain barrier to chemo-therapeutics (Khaitan et al., 2018). Kir6.2 upregulation has been found in mito-KATP channels in some tumor cell lines (Hons and Singh, 2005).

Previous clinical literature data have shown that the expression of *ABCC8* was found downregulated in pancreatic cancer (Mohelnikova-Duchonova et al., 2013), triple-negative breast cancer (Hlaváč et al., 2013), and lung adenocarcinoma (Wang et al., 2020); the low expression of *ABCC8* was associated with poor prognosis in these tumors, while high expression with improved overall survival O.S. In line with these data, *ABCC8/*Sur1 has been proposed as a new independent prognostic index for glioma patients that had longer survival (Zhou et al., 2020). Upregulation of *ABCC8* was also found in brain tumors (Huang et al., 2009; Ocampo-Garza et al., 2019) and gastric cancer (Mao et al., 2019) and downregulation in colon–rectal cancer (CRC) (Hlavata et al., 2012). Mutations were found (Xiao et al., 2019) in patients affected by pancreatic neuroendocrine tumors (PanNETs); genetic variations are also present in hepatoblastoma and breast cancer (Calton et al., 2013; Soucek et al., 2015). However, the role of the sulfonylureas in cancers in diabetic type II patients remains controversial (Hendriks et al., 2019).

The role of the *ABCC9/*Sur2 gene in cancers is not described, and it is controversial*.* The *ABCC9* and *KCNJ11* genes were found upregulated in cervical cancer (Vázquez-Sánchez et al., 2018); the *ABCC9* gene also in gastric cancer (Mao et al., 2019) and epithelial ovarian cancer (Elsnerova et al., 2017). Downregulation of the *ABCC9* gene was found in triple-negative breast cancer (Zhang et al., 2020) and prostate cancer (Demidenko et al., 2015). Mutations of the *ABCC9* gene were reported in large granular lymphocyte leukemia (Cheon et al., 2022), endometrial (Le Gallo et al., 2012), gastric (Zhang et al., 2022), and breast cancers (Ivan et al., 2021).

Additionally, the *KCNJ8* and *KCNJ11* genes were, respectively, found upregulated in the adenocarcinoma of the esophagus (Warnecke-Eberz et al., 2016) and hepatocellular carcinoma (Zhang et al., 2018).

Two patients were carrying missense variants in the *ABCC9/*Sur2 gene with acromegalic features and hypertrichosis, but the normal growth hormone (GH) axis also had clinically non-functioning pituitary macroadenomas, a feature which has not previously been associated with C.S. C.S. is associated with gain-of-function (G.O.F) mutations of *ABCC9* and *KCNJ8* genes. Interestingly, in minoxidil (MXD) treatment, mice and rats are considered pharmacological models of C.S. sharing a similar cardiovascular phenotype. There is no clear explanation why activating this channel would lead to adenoma (Marques et al., 2018b; 2018a). Minoxidil is a well-known opener of KATP channels targeting the Sur2 subunit used in hypertension and extensively used in alopecia.

We recently reported that renal cancer was one of the most serious adverse reactions (A.D.Rs.) observed in male rats under topical sub-chronic treatment with high dosing of minoxidil formulation (Tricarico et al., 2018; Maqoud et al., 2020) and cancer reactions in human at a therapeutic dose for the treatment of androgenic alopecia (Hsu et al., 2014). Mammary adenomas and adenocarcinomas were found in female mice and pheochromocytomas in both genders in rats following treatment with topical MXD for 90 days (Product monogram ROGAINE^®^, 2017).

Therefore, it seems that the gene upregulation or the drug-induced overactivation of the KATP channel subunits, specifically Sur2A and its accessory subunits Kir6.1 and/or Kir6.2, can be associated with cancers.

In contrast, a recent report showed that minoxidil treatment is effective in reducing tumor growth in an animal model of ovarian cancer, and following a series of bioinformatics analyses, the authors demonstrated that among the four genes encoding for the KATP channel subunits, the *ABCC9/*Sur2 subunit is downregulated in ovarian cancer and the upregulation of this gene is a positive prognostic factor in woman in this cancer type (Fukushiro-Lopes et al., 2020). Considering the aforementioned data reported, the role of the Sur2A subunit and its accessory subunits Kir6.1 and Kir6.2 in cancer requires investigation.

In the present work, through an experimental immunohistochemical study, we investigated the role of the Sur2A subunit and its accessory subunits in renal cancer observed in male rats sub-chronically and topically treated with a high dosage of minoxidil hydroalcoholic formulation (0.777–77.7 mg/kg/day), and the role of the Sur2A and Kir6.1-2 subunits in canine biopsies of female breast cancer that is used as a spontaneous animal model of disease.

Also, pharmacovigilance data were analyzed to assess the correlation of *ABBC8*, *ABCC9*, *KCNJ11*, and *KCNJ8* genes with cancer reactions induced by drugs targeting the different KATP channel subunits.

The relationship between these genes and cancers and the effects of race and gender were investigated by omics analysis.

## Materials and Methods

### Animal treatment and immunohistochemical analysis on animal samples treated with high dosing of minoxidil solution

The animal treatment was carried out according to Italian Legislative Decree No. 26 of 04 March 2014 and performed under the supervision of a local veterinary official. Using an operator-blinded design, the animals were randomly assigned to each experimental condition as previously described (Maqoud et al., 2020). The Organization for Animal Health of the University of Bari on 15 April 2019 (Prot. 34,091-x/10, 06 May 2019) and the Minister of Health, Rome (No. 673/2019-PR Prot. DGSAF0025334-P-04/10/2019, prog. 7307D.11), approved the protocol.

The experiments were performed on male Wistar rats (Charles River S. p.A., Calco, Lecco) with a mean body weight of 360 ± 14 g that were treated with minoxidil solutions and relative vehicles (number of rats = 10). A repeated escalating dose of 0.035% (0.777 mg/kg/day), 0.07% (1.554 mg/kg/day), and 3.5% (w/v-w) (77.7 mg/kg/day) of minoxidil (MXD) ethanol/glycol propylene solution (number of rats = 5) was topically applied to the rats; other rats were treated with vehicles (Maqoud et al., 2020). The cumulative dose of MXD at the end of the treatment period was 71.15 g/kg.

At the end of treatment, the animals were killed by the overdose of the anesthetic agent Zoletil 50/50 (Pfizer) i. p. 60 mg/kg, followed by cervical dislocation, and the tissue samples such as the heart, liver, kidneys, skin, and muscles, but in this work, we investigated the kidney, were immediately preserved in 10% buffered formalin. The EDTA blood and serum were processed. The histological examination was performed for each animal sample ≥ 48 h after fixation. The tissues and organs were embedded in paraffin, cut into 5 μm sections, and stained using standard techniques with hematoxylin and eosin. The histopathology and necropsy analysis included a macroscopic and microscopic examination of the tissue samples and an organ assessment of the preserved kidneys and other tissues. Finally, an examination of the cellular morphology, as well as the relationship between the nucleus, nucleolus, and cytosol was conducted. A histopathological scoring system, based on the semiquantitative ordinal system, was used to evaluate the severity of the observed lesions. The pathologists were blinded to the treatment of the analyzed samples. Images from 10 random fields were acquired for the 10 stained sections of each specimen using a D 4000 Leica DMLS microscope equipped with a camera and image analyzer, NIS-Elements BR (Nikon). The analysis of the sections was performed using Leica QWin software. Organ weight was not measured.

The autopsy of the various organs revealed the congestion of all the parenchyma. The kidneys on external examination showed no modifications, only in three subjects did the cut surface show small pale foci with a maximum size of 0.2 cm in diameter, and the remainder showed no significant alterations. The dissected kidneys were fixed in 10% buffered formalin, dehydrated, and subsequently embedded in paraffin, sectioned to 4 microns, and stained with hematoxylin and eosin (H&E), periodic Schiff acid (PAS), and tricolor by Masson for routine diagnostics. The diagnostic criteria used followed the guidelines published by the Society of Toxicologic Pathology (Hard and Seely, 2005; Hard and Seely, 2006), and the proliferative lesions of the tubules were reported by Hard and Seely (2005, 2006) and Hard et al. (2008).

We performed immunohistochemical staining of the kidney sections with the avidin–biotin streptavidin (LSAB) method using the OMNIS Immunohistochemistry Staining System, Agilent Technologies.

The slides were controlled by protocols using DakoLink software. Tissue sections were cut (4 μm thick), placed on poly-L-lysine-coated slides, xylene de-waxed, and dehydrated. IHC slides were automatically mounted, and coverslips were applied, after staining and dehydration. To detect the antigens, the sections were immersed in citrate buffer (0.1, pH 0.6) for 30 min with 0.3% hydrogen peroxide and then in methanol for 12 min to quench the peroxidase activity. After washing three times for 5 min each with phosphate-buffered saline (PBS), the sections were blocked by soaking them for 20 min at room temperature in PBS containing 1% bovine serum albumin. After rinsing with PBS, the blocked sections were incubated overnight at 48°C, and the sections were stained with polyclonal and/or monoclonal antibodies (Table 1).

**TABLE 1:** The immunohistochemical markers were used in the kidney cancer in the rat.

**Antibody**	**Clone**	**Dilution**	**Company**
Cytokeratin 8/18 M	EP17/EP30	1/100	Dako, Glostrup, Denmark
Vimentin M	V9	1/100	Dako, Glostrup, Denmark
CD117, c-kit M	104D2	1/100	Dako, Glostrup, Denmark
CD10 M	56C6	1/100	Dako, Glostrup, Denmark
CD44 M	DF1485	1/100	Dako, Glostrup, Denmark
P53 M	DO-7	1/100	Dako, Glostrup, Denmark
Calponin M	CALP	1/100	Dako, Glostrup, Denmark
E-Cadherin M	NCH-38	1/100	Dako, Glostrup, Denmark
Ki-67 M	MIB-1	1/100	Dako, Glostrup, Denmark
Anti Kir6.1/ *KCNJ8* P	ab241996	1/50	Abcam, Cambridge, United Kingdom
Anti-Kir6.2 / BIR anti *KCNJ11* P	AB79171	1/50	Abcam, Cambridge, United Kingdom
Anti-*ABCC9*/SUR2A M	AB174629	1/300	Abcam, Cambridge, United Kingdom
Anti *ABCC8*/SUR1 M	ab134292	ab134292	Abcam, Cambridge, United Kingdom

M, monoclonal; P polyclonal.

Positive controls were inserted for each type of antibody during the immunohistochemical procedure using already tested tissue samples of organs as suggested by the datasheets. For the negative control, the primary antibody was omitted during immunohistochemical staining. The slides were controlled by protocols using the DakoLink software.

The antibodies were visualized using a biotinylated secondary antibody, an avidin–biotin–peroxidase complex, and 3-amino-9-ethyl carbazole as chromogens (Dako, Glostrup, Denmark). Nuclear counterstain was performed with Gill’s hematoxylin (Polysciences, Warrington, PA, United States), and then, the sections were dehydrated with ethanol and xylene before assembly. The sections were initially examined with a magnification of × 200 (i.e., objective × 20 and eyepiece lens × 10; 0.7386 mm^2^ per field) and subsequently to × 400 × fields (i.e., objective × 40 and eyepiece lens × 10; 0.1885 mm^2^ per field). Images of all sections were captured using an HD camera (Nikon Corporation, DS-Fi2 high-definition color camera, Tokyo, Japan) connected to an optical microscope (Nikon Corporation, Nikon Eclipse Ni-U, Tokyo, Japan) with 20x–40x objective; morphometric analyses were performed using an interactive image analysis system based on Arivis Vision4D, modular software for working with multi-channel 2D, 3D, and 4D (Nikon Corporation, NIS-Elements BR, Tokyo, Japan).

Appropriate positive and negative controls were included at each immunohistochiemical (IHC) run. Cancer cells were considered only positive when the appropriate staining patterns for the staining have been achieved. The extent of the immunohistochemical reaction of tumor cells was classified into levels, such as no positive tumor cells (0); positive cells < 10% (1+) corresponding to G1, positive cells 10%–50% (2+) = G2, and positive cells> 50% (3+) = G3 according to the guideline (Zizzo et al., 2019). A defined score from 0 to 1+ was given as negative and scores 2+ and 3+ as positive. Evaluation of the samples was performed independently by three of the authors (NZ, GP, and AT). Positive staining for a given marker was considered only when all the observers agreed on its specificity and distribution.

### AgNOR staining

Additional kidney sections in rats were stained with an argyrophilic technique that assesses the NOR (region of the nucleolar organizer) (Dako, Glostrup, Denmark). Briefly, the sections were de-waxed and incubated with the AgNOR solution in a dark room for 45 min. After washing with distilled water, the sections were incubated in alcohol for 4 min and in xylene for 3 min and then mounted with a mounting medium. The number of nuclear points (AgNOR) from 100 cells was counted using a magnification of 1.000 x (oil immersion). The mean AgNOR score per cell was expressed as the AgNOR score.

### Animal treatment and immunohistochemical experiments on canine female breast cancer samples

A total specimen from 23 female dogs with spontaneous mammary neoplasia has been recruited into the archives of Pathology and Comparative Oncology to the Department of Medicine Veterinary (Bari-Italy) for diagnosis with the written consent of the animal owners and ethical approval from the University of Bari (Zizzo et al., 2019; Tricarico et al., 2022). The mammary glands were removed with radical mastectomy or partial, with regional lymph nodes. Surgical samples fixed in 10% buffered formalin and stained with hematoxylin–eosin were classified histologically following the Goldschmidt classification (Goldschmidt et al., 2011) and Peña for the tumor grading system (Patruno et al., 2009; Zizzo et al., 2010; Peña et al., 2013; Rasotto et al., 2017). The sections were cut (4 μm thick), placed on poly-L-lysine-coated glass slides, and subsequently, deparaffinized in xylene and dehydrated and immunohistochemically stained according to the labeled streptavidin avidin–biotin (LSAB) method (Ioachim, 2008) using antibodies in Table 2.

**TABLE 2:** Immunohistochemical markers used in the female canine breast cancer.

Ki-67
Anti Kir6.1 / *KCNJ8*
Anti Kir6.2 / BIR *KCNJ11*
Anti-*ABCC9*/SUR2A
Anti *ABCC8*/SUR1

Dilution and data related to the company providers and mAbs were reported in Table 1.

Analysis of the canine sections was performed as previously described and reported (Ioachim, 2008; Patruno et al., 2009; Zizzo et al., 2010; Goldschmidt et al., 2011; Rasotto et al., 2017; Tricarico et al., 2022).

### Omics analysis

A large amount of genomic data concerning pathological and polymorphic variants in various tissues in both humans and animals, phenotypic features, bibliographic documents, alternative transcripts, gene expression, and other data derived from omics studies are available through databases implemented on the NCBI Entrez system (https://www.ncbi.nlm.nih.gov/). Currently, we have focused our preliminary research on systematic and integrated navigation of the various bioinformatic resources starting from the Gene database (Brown et al., 2015). Here, detailed analyses were reported for data regarding gene expression and variation.

Moreover, starting from PubMed data reported in the Gene database and through this, in OMIM, we have selected publications where the terms cancer and tumor have been mined.

### Mining cancer-related articles in PubMed

A protocol allowing the retrieval of information available in the literature concerning the four genes *ABCC8*, *ABCC9*, *KCNJ11*, and *KCNJ8* here was described taking as an example the *ABCC8* gene. Starting from the NCBI site and browsing the available databases (35 at the time of the research) for searching data regarding *ABCC8*, non-null results are available in 29 databases. The protocol has been performed to start from the PubMed dataset available through Bibliography/Related articles in PubMed/see all articles (q1 = #1) and from the PubMed OMIM dataset (q1 = #7) available through the *Related data* from the gene entry. The terms considered are “cancer” and “tumour,” both searched in all fields (queries #2,#3,#8, and #9) and in *MesSH Term*s (queries #4, #5, #10, and #11). The application of the OR operator for #2, #3, #4, and #5 and for #8, #9, #10, and #11 has produced the lists of our interest derived from q1 (i.e., q6 associated to 21 papers) and q7 (i.e., q12 associated to 22 papers). Finally, applying the OR operator to q6 and q12 has produced the list q13 associated with 38 papers (that were further analyzed). The same protocol was reproduced for the other three genes, *ABCC9*, *ABCC9*, *KCNJ11*, and *KCNJ8* genes.

We have focused on the sections Variation, Bibliography, and Expression. The section Bibliography reports links to Related articles in PubMed (the list of papers selected by the curators of the Gene database) and GenRif (short notes reported as communication by registered users). Thus, by clicking on “Bibliography/Related articles in PubMed/see all articles,” the user is automatically migrated from Gene to PubMed where a list of 274 papers is available. However, because the need is to choose papers reporting data regarding oncological studies, the advanced query procedure is activated based on the selection of the term “cancer” as free text or as a mesh term combined with list 1; according to the usage of logical operators, a list of 25 papers has been obtained.

### Gene expression

The analysis of gene expression data produced through both microarrays and RNA-seq technology has been performed thanks to the availability at the NCBI of the Human Protein Atlas (HPA) database (Fagerberg et al., 2014) (BioProject PRJEB4337, DOI: 10.1074/mcp.M113.035600) annotating RNA-seq data of 27 different tissues samples from 95 human individuals to determine the tissue specificity of all protein-coding genes. The four genes’ analysis has been also performed by browsing the Expression Atlas database (Papatheodorou et al., 2020) (https://academic.oup.com/nar/article/48/D1/D77/5609521), where data regarding samples recruited within the Pan Cancer project (https://www.embl.de/campaigns/pancancer/) and whose expression has been estimated are annotated and available at the EBI (https://www.ebi.ac.uk).

### Differential gene expression in normal and cancer samples

The availability of data regarding both normal and disease-associated samples allows for estimating the differential expression of the same gene. At this aim, the TNMplot web tools, a web tool for comparing gene expression in normal, tumor, and metastatic tissues, were considered. The TNMplot.com web tools are based on data generated from gene arrays from the Gene Expression Omnibus of the National Center for Biotechnology Information (NCBI-GEO) or RNA-seq from The Cancer Genome Atlas (TCGA), Therapeutically Applicable Research to Generate Effective Treatments (TARGET), and the Genotype-Tissue Expression (GTEx) repositories. Statistical significance was calculated using Mann–Whitney or Kruskal–Wallis tests. The entire database contains 56.938 samples, including 33.520 samples from 3.180 genetic chip-based studies (453 metastatic, 29.376 tumor, and 3.691 normal samples), 11.010 samples from TCGA (394 metastatic, 9.886 tumors, and 730 normal), 1.193 samples from TARGET (1 metastatic, 1.180 tumor and 12 normal), and 11.215 normal samples from GTEx. The tumor tissues of gene expression data that are considered in TNMPlot are adrenal cancer, acute myeloid leukemia (AML), bladder cancer, breast cancer, colon cancer, esophageal carcinoma, liver cancer, lung adenocarcinoma, lung squamous cell carcinoma, ovarian serous cystadenocarcinoma, pancreatic adenocarcinoma, prostate adenocarcinoma, rectum adenocarcinoma, renal clear cell carcinoma, kidney chromophobe, kidney renal papillary cell carcinoma, skin cutaneous melanoma, testicular germ cell tumors, thyroid carcinoma, uterine carcinosarcoma and uterine corpus, and endometrial carcinoma. The statistical significance of the differential expression is calculated by applying a Mann–Whitney *U* test.

### TNMplot.com/kmplot.com/webtools

Pan Cancer’s RNA-seq data were analyzed by selecting the section gene expression comparison on paired tumor and adjacent normal tissues. The research was carried out for every single gene in the different tissues through the use of the TNMplot.com web tools.

The TNMplot.com web tools, a web tool for comparing gene expression in normal, tumor, and metastatic tissues, are used. The TNMplot.com web tools are based on data generated from both gene arrays from the NCBI-GEO or RNA-seq from TCGA, TARGET, and the GTEx repositories. The entire database contains 56.938 samples, including 33.520 samples from 3.180 genetic chip-based studies (453 metastatic, 29.376 tumor, and 3.691 normal samples), 11.010 samples from TCGA (394 metastatic, 9,886 tumors, and 730 normal), 1.193 samples from TARGET (1 metastatic, 1.180 tumor and 12 normal), and 11.215 normal samples from GTEx. The research was carried out for every single gene in the different tissues (Le Gallo et al., 2012; Soucek et al., 2015; Cheon et al., 2022; Zhang et al., 2022).

Instead, via kmplot.com/webtools, considering the Pan Cancer projects, the dataset of RNA-seq samples with follow-up comprised 9,663 specimens from 26 distinct tumor types. Across the entire database, the median follow-up for overall survival (OS) was 24.3 months, and for relapse-free survival (RFS), it was 23.8 months. Correlations between gene expression and survival were calculated using Cox proportional hazards regression. The correlation search was performed on the www.kmplot.com database, using the gene name, selecting trichotomization (Q41vs. Q4: lower quartile vs. upper quartile), dividing the search by gender, using the race restriction, and selecting only whites (Győrffy, 2021).

Statistical significance was calculated using Mann–Whitney or Kruskal–Wallis tests.

### Pan Cancer survival analysis

The correlation between gene expression and overall survival (OS) was made using Cox proportional hazard regression analysis. Package R “survival” v2.38 (http://CRAN.R-project.org/package=survival/) was used to calculate log-rank *p*-values, hazard ratios (HRs), and confidence intervals at 95% (CI). Furthermore, survival differences were visualized by generating Kaplan–Meier survival plots.

Grouping and hierarchical clustering were applied to visualize the characteristic cancer genes associated with survival in different cancer types using the Genesis software.

### Pharmacovigilance analysis

All coding preferred terms per S.O.C. were screened in the EudraVigilance database (http://www.adrreports.eu/), and those potentially associated with KATP channel actions were reported and subject to analysis. The data were extracted from EudraVigilance, collected, and analyzed on Excel software (Microsoft 10.00). The data were collected per age and sex. The duplication of a specific report per system organ class (S.O.C.) was evaluated manually and excluded from the analysis (Mele et al., 2014).

### Statistical analysis

The data were reported as an average ± E.S. unless otherwise specified. The significance between data pairs was calculated by paired Student’s t-test for *p* < 0.05. One-way ANOVA was used to evaluate significance within and between data with variance ratio F > 1 at significant levels of *p* < 0.05.

The proportional reporting ratio (P.R.R.) was calculated using the equation a/(a+c)/b/(b + d), where a is the reaction of interest to a given drug of interest, b is the reaction of interest for all other drugs in the class, c are all other reactions to a given drug of interest, and d represents all other reactions to all other drugs in the class. Signal definition: P.R.R. ≥ 2, a minimum of three ratios/cases for the reaction of interest, X^2^ ≥ 4. No signal was identified if P.R.R. is = 1.

The IC_50_ data of different KATP channel blockers were pooled from the literature and were obtained from concentration–response curves of transmembrane KATP channel currents vs. at least five to seven different drug concentrations. The IC_50_ values were derived from the standard formula and reflect the drug concentration that would inhibit 50% of the KATP channel current when measured in a drug-free solution and expressed as fractional current. The data were obtained in a variety of cell lines and sources for Sur and KATP channel proteins to obtain the IC_50_ values from different laboratories as previously reported (Abdelmoneim et al., 2012; Maqoud et al., 2016).

Correlations between gene expression and survival were calculated using Cox proportional hazard regression and plotting Kaplan–Meier survival plots. The correlation search was performed on the www.kmplot.com database, using the gene name, selecting trichotomization (Q41 vs. Q4: lower quartile vs. upper quartile), dividing the search by gender, using the race restriction, and selecting only whites. RNA-seq data were reported in RPKM (reads per kilobase million) or FPKM (fragments per kilobase million) and TPM (transcripts per kilobase million).

## Results

### Minoxidil-induced renal cancer in male rats

The long-term topical treatment with high dosing of the minoxidil ethanolic solution induces cell proliferation and cancer in male rats. All five of the rats treated with the MXD ethanol/glycol propylene solution showed lesions in all the organs sampled. In the repeated escalating doses of the MXD (0.035%–3.5% w/v-w) experiment, the rats treated once daily with either of the MXD at concentrations of 0.035% (0.0777 g/kg/day), 0.07% (0.1555 g/kg/day), and 3.5% (w/v-w) (7.777 g/kg/day) for 2 months experienced mild and insignificant reductions in their arterial blood pressure with no change in HR, shown by telemetry monitoring in free-moving rats (Maqoud et al., 2020). The cumulative dose of MXD that the experimental groups received using this protocol was 71.15 g/kg. Renal cancer was not fatal in these rats, and no reduction in body weight or other clinical signs were observed.

**FIGURE 1:**
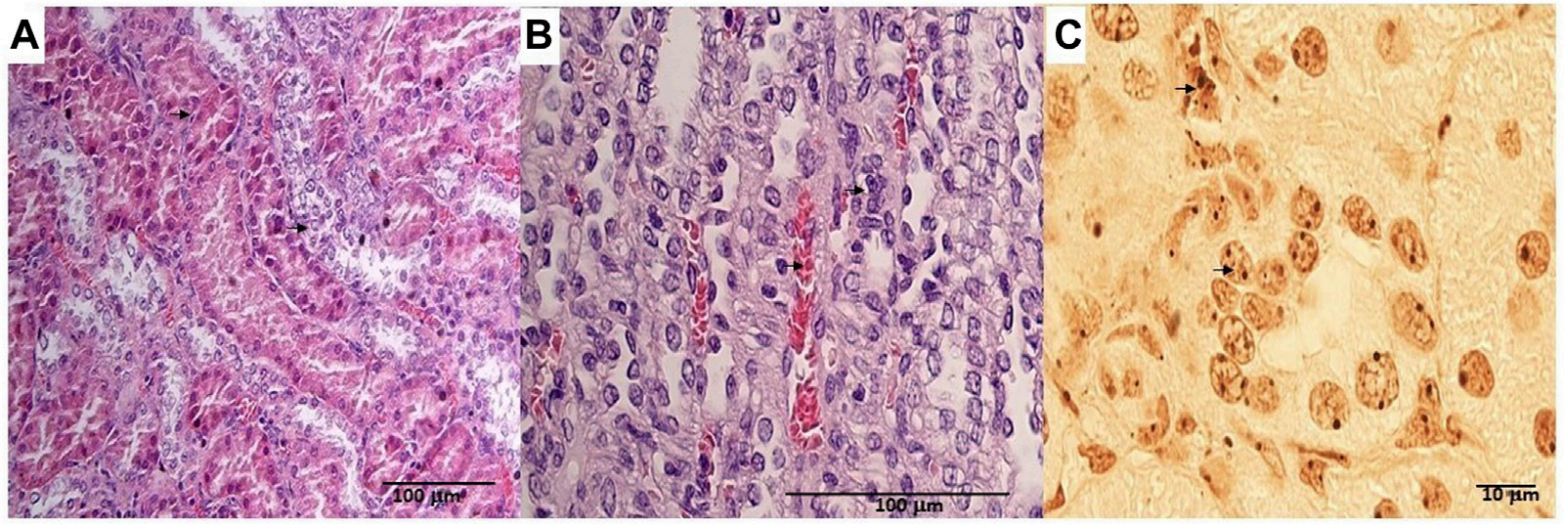
**(A)** Degenerated proximal and distal convoluted tubules, thickened basement membrane, a clear nucleus with karyomegaly (up arrow), and prominent or absent nucleoli in the rat tumor renal section (down arrow). H.E. bar = 100 μm, 20X. **(B)** Tumor apical portion of the pyramid: tubules and papillary ducts have cells arranged randomly, cell pleomorphism, high nucleus/cytoplasm ratio (up arrow), prominent nucleoli, and discrete mitosis, rich vascularized (down arrow). H.E. bar = 100 μm, 40X. **(C)** AgNor staining of rat renal tumor cells showing prominent nucleus in single cells (arrow). Bar = 10 μm, 10X.

**FIGURE 2:**
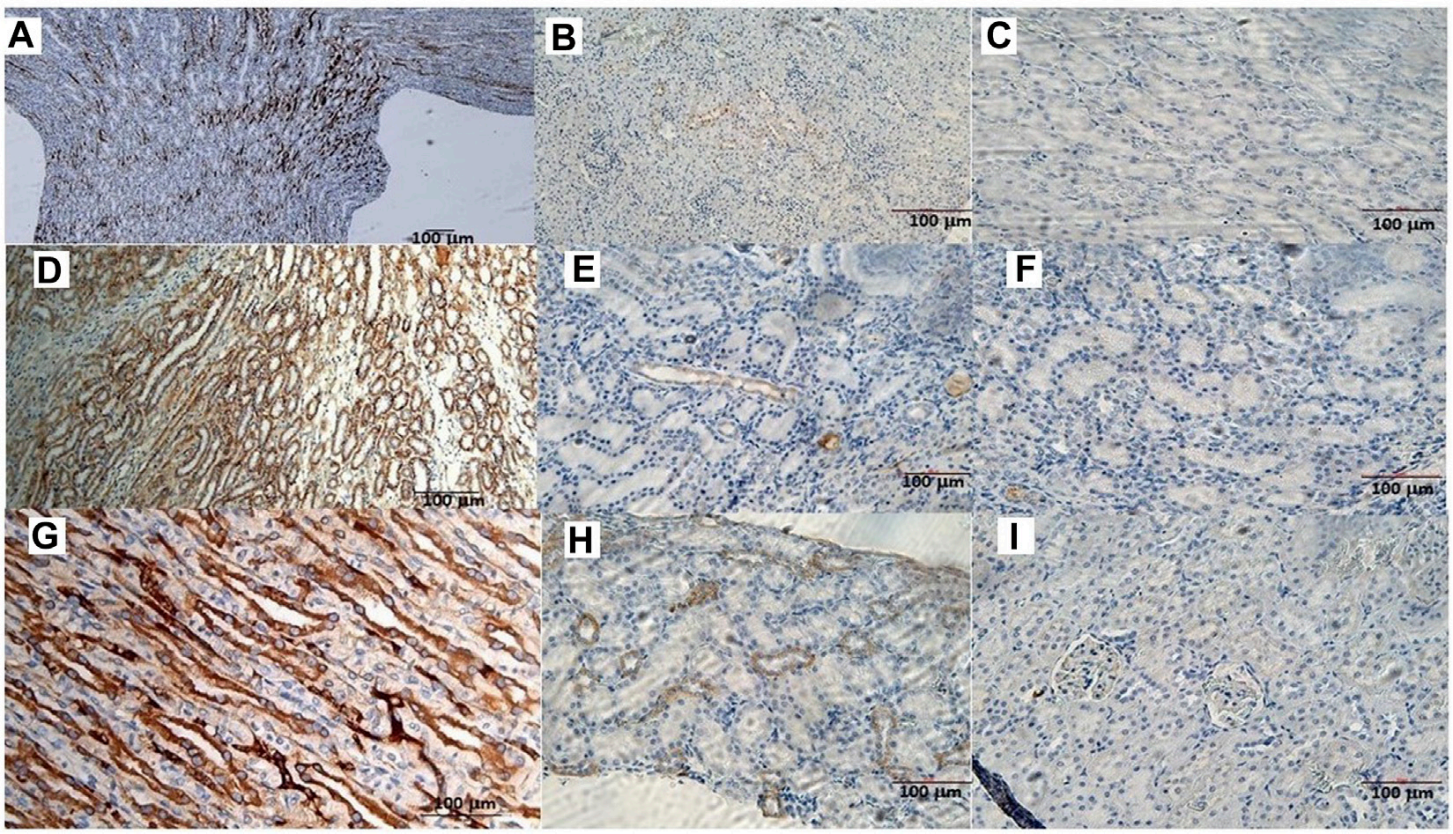
**(A)** CD117 positive neoplastic cells demonstrating strong diffuse cytoplasmic staining in the rat tumor renal section. Bar = 100 μm, 5X. **(B)** CD117 reaction in a rat renal section h used as a positive control. Bar = 100 μm, 10X. **(C)** CD117 negative control in the absence of mAb anti-CD117. Bar = 100 μm, 20X. **(D)** Cell membrane staining positive for the E-cadherin antigen. Bar = 100 μm, 10X. **(E)** E-cadherin positive section. Bar = 100 μm, 20X. **(F)** E-cadherin negative section. Bar = 100 μm, 20X. **(G)** The cytoplasm of the epithelial cells forms tubules positive for cytokeratin antigen staining. Bar = 100 μm, 20X. **(H)** Cytokeratin positive section. Bar = 100 μm, 20X. **(I)** Cytokeratin negative section. Bar = 100 μm, 20X.

**FIGURE 3:**
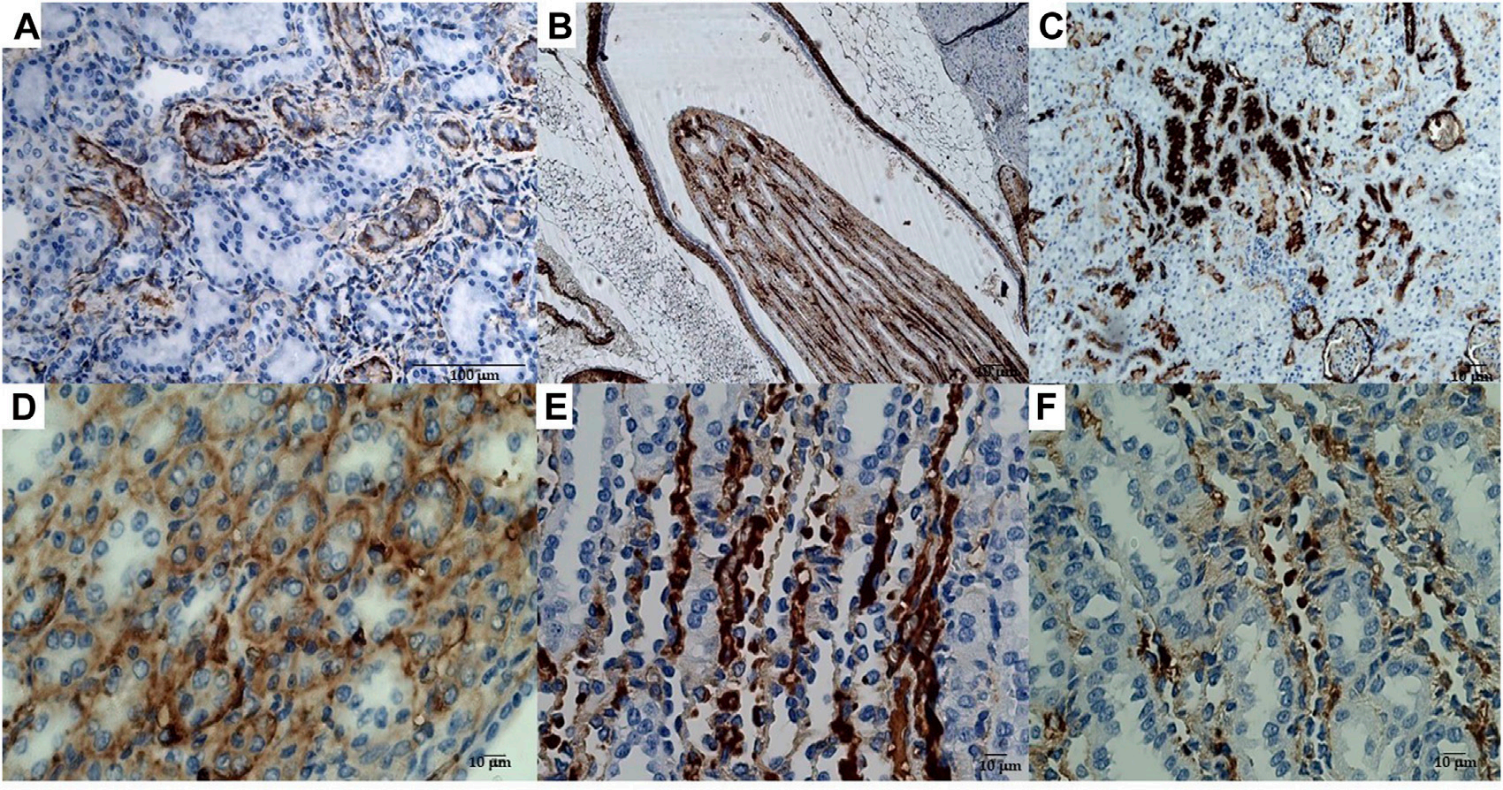
**(A)** Immunohistochemical reactions of the rat kidney tumor sections to mAbs: **(A)** vimentin, bar = 100 μm, 20X; **(B)** calponin, bar = 10 μm, 10X; **(C)** CD10, bar = 10 μm, 10X; **(D)** CD44, bar = 10 μm, 40X; **(E)** Ki67, bar = 10 μm, 10X, and **(F)** p53, bar = 10 μm, 40X.

The animals were killed after treatment. The *postmortem* renal biopsy from the MXD-treated animals showed degenerated proximal and distal convoluted tubules, thickened basement membrane, and a clear nucleus with karyomegaly, and prominent or absent nucleoli were indicators of rat tumor renal section. Also, the tumor apical portion of the pyramid showed tubules and papillary ducts with cells arranged randomly, cell pleomorphism, high nucleus/cytoplasm ratio, prominent nucleoli, and discrete mitosis in the renal section following H.E. staining from three rats (Figures 1A, B). All cases are reported in Table 3. On the other hand, for AgNor staining, two to four positive regions of the nucleolar organizer (NOR) granules were identified in the nuclei of the cells of the distal tubules and the ascending flap of the loop of Henle of the kidney in subjects with neoplasia (Figure 1C). Immunohistochemical staining of the kidney section with Ki67 was performed to evaluate the proliferative status of the cells, and we found that 85.2% ± 1% of the cells in the analyzed sections were positive for the antibody reaction following treatment with the MXD ethanol/glycol propylene solution in the higher dosing group. These sections also showed an elevated reaction to CD117 (Figures 2A–C), E-cadherin (Figures 2D–F), and cytokeratin (Figures 2G–I) cells which showed 10%–50% positive cells vs. positive controls. The sections of the tissues were all positives to vimentin, calponin, CD10, CD44, Ki67, and p53 (Figures 3A–F) vs. non-cancerous renal tissues.

**TABLE 3:** Case reports of renal cancer in rats treated with high doses of minoxidil ethanol/glycol propylene solution and severity grading.

N=rats/organ	Type of injury	Description	MXD ethanol/glycol prop. *N* rats = 5	Ethanol/glycol prop *N* rats = 5
Five MXD rats/dx, sx kidney	Circulation disorders	Congestion	**++**	**+**
	Nephrosis	Glomerulo/tubulo nephrosis	**+++**	**+**
	Nephritis	Glomerulo/tubulo interstitial nephritis	**++**	**+**
Three MXD rats/dx, sx kidney	Neoplasia	Pale foci with a maximum size of 0.2 cm in diameter, degenerated tubules, thickened basement membrane, nucleus with karyomegaly, high nucleus/cytoplasm ratio, prominent nucleoli, mitosis, rich vascularization	**+++ (G3)**	**+**
		Ki67 = 85		

**FIGURE 4:**
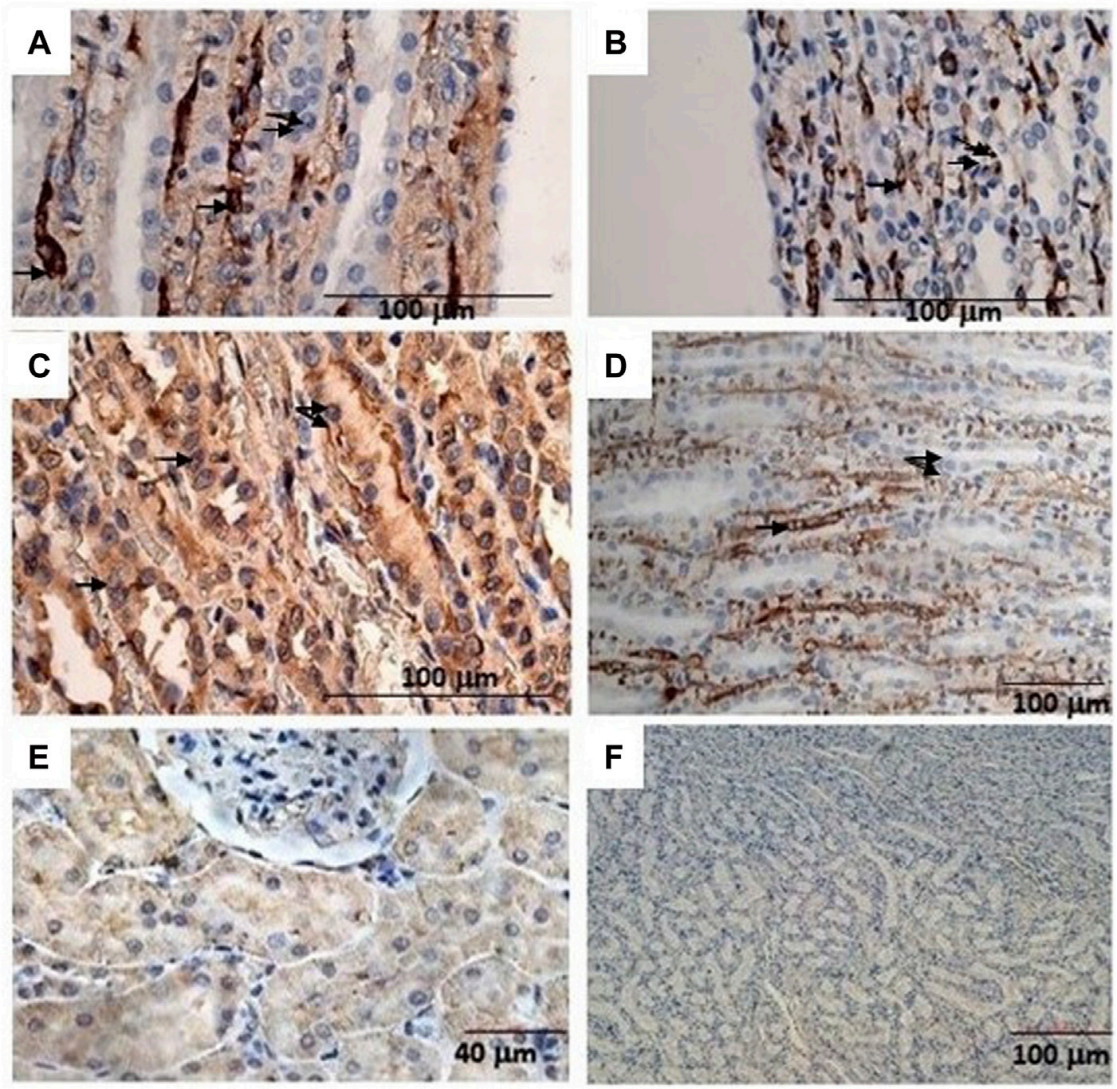
**(A)** Sur1 immunohistochemical detection localized in the cytoplasm and plasma membrane of renal cancer cells; double arrows indicate normal cells, and a single arrow indicates abnormally stained cells. Bar = 100 μm, 40X. **(B)** Kir6.1 immunohistochemical detection localized in the basal areas of cells (single arrow), normal cells (double arrows). Bar = 100 μm, 40X. **(C)** Abnormal Sur2A immunohistochemical detection localized in the cytoplasm and plasma membrane showing > 50% of positive cells vs. control. Bar = 100 μm, 40X. **(D)** Kir6.2 immunohistochemical localized in the basal areas of cells was found (single arrow) and in normal cells (double arrows). Bar = 100 μm, 10X. **(E)** Typical immunohistochemical staining with the primary mAb in the cytoplasm of the tubules used as a positive control for Sur2A in a non-cancer rat kidney section. Bar = 40 μm, 40X. **(F)** A rat renal section with no primary mAb was used as a negative control. Bar = 100 μm, 10X.

The immunohistochemical reaction against the Sur2A subunit (Figure 4C) was markedly elevated in the cytosolic compartment in CD117, E-cadherin, and cytokeratin cells showing 10%–50% positive cells vs. positive controls (Figures 4C, E, F), and a less marked immunohistochemical reaction is observed in the other subunits in the cancerous cells (Figures 4A, B, D). The immunohistochemical panel used allowed us to highlight a different percentage of immunohistochemical reactivity in the various structures of the renal parenchyma in subjects with neoplastic and non-neoplastic lesions (Table 1).

Therefore, high-dose administration of minoxidil induced cell proliferation in renal Ki67-positive cells and the upregulation of the Sur2A located in the cytosolic compartment in renal cancer cells in male WT rats (Figure 4C) vs. controls (Figure 4E).

### Immunohistochemical reaction of the Sur2A subunit in the cytosolic compartment of the G3 cells of canine breast cancer

**FIGURE 5:**
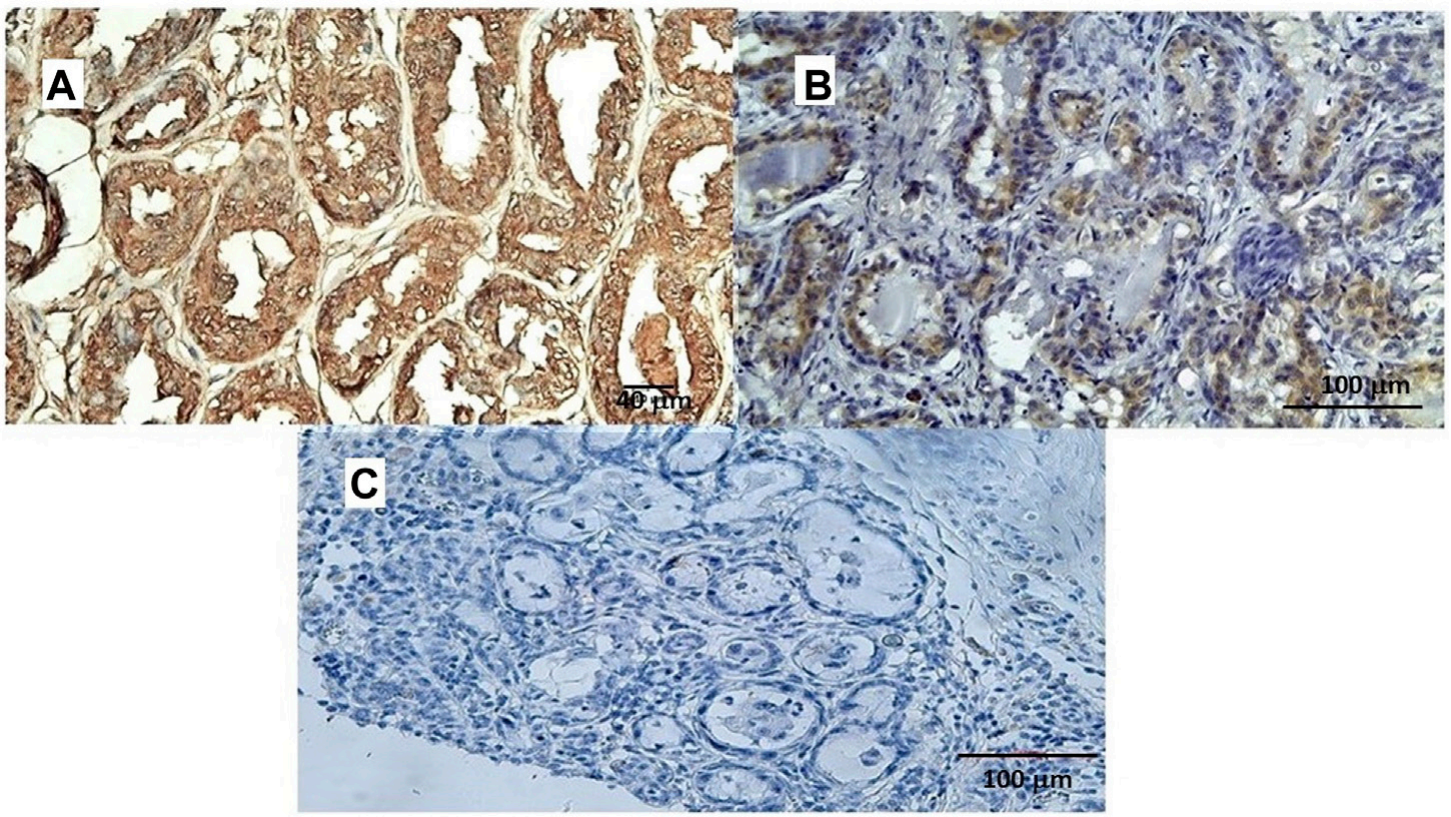
**(A)** Sur2A immunohistochemical reaction localized in the cytoplasm and plasma membrane of canine female breast cancer cells in G3. Bar = 40 μm, 20X, showing > 50% of positive cells vs. control. **(B)** Sur2A immunohistochemical detection in a section used as a positive control. Bar = 100 μm 20X. **(C)** Section of tissue in the absence of mAb anti Sur2A used as a negative control. Bar = 100 μm 20X.

In a study conducted on samples of spontaneous mammary neoplasia in purebred and mixed-breed dogs aged between 4 and 15 years (average 9.5), not subjected to chemotherapy treatments, from 2015 to 2019 in various veterinary clinics in Bari, Italy, biopsies were collected after tumor removal surgically from female dogs by radical mastectomy or regional mastectomy, with inguinal lymph node removal (Zizzo et al., 2019). Immunohistochemical reaction investigations showed that the Sur2A subunit was markedly elevated in the cytosolic compartment in G3 cells (Figures 5A–C) vs. positive controls, and no changes were observed for G1 or G2 cells; in contrast to the Sur2A subunit for the other subunits, no marked reaction was observed in cancerous cells. The tumor samples (N cases = 23) were classified as the carcinoma samples: tubular, tubule-papillary, and cystic-papillary as previously reported (Zizzo et al., 2019).

Therefore, the cytosolic immunohistochemical reaction against Sur2A h was found in two different cancers in distinct species supporting the involvement of Sur2A in these cancer types. We, therefore, tested the role of the KATP channels in the pharmacovigilance database and omics databases.

### Pharmacovigilance investigations

Pharmacovigilance data were analyzed to assess the possible correlation between KATP channels and adverse cancer reactions induced by drugs targeting KATP channels. Several cases of cancers were observed with KATP channel blockers used in the treatment of type II diabetes. The most commonly reported were pancreatic carcinoma, malignant neoplasm, and myelodysplastic syndrome followed by bladder cancer and some cases of hepatocellular, breast, prostate, colon, and renal cancers (Table 4).

**TABLE 4:** Adverse drug reactions (A.D.R.) of KATP blockers and openers reported under the System Organ Class (S.O.C.): Neoplasm benign, malignant, and unspecified disorders and preferred terms P.T. The not-specified category was not included in the present table. Data were pooled from Eudra Vigilance database.

Number of A.D.R. of KATP channel blockers and openers in female (F) and male (M)	Neoplasm benign, malignant, and unspecified disorders
Glimepiride 6432: 2953F, 3062M	138: pancreatic carcinoma 19, not resolved 2, fatal 4; myelodysplastic syndrome 7; neoplasm malignant 7; bladder cancer 7; prostate cancer 6; colon cancer 5; breast cancer 3; gastric cancer 3; skin cancer 3; hepatocellular carcinoma 2; lung malignant neoplasm 2; renal cell carcinoma 2; renal cancer 1; cervix carcinoma 1.
Glibenclamide 3815: 1751F, 1841M	49: pancreatic carcinoma 5; bladder cancer 3; breast cancer 3; hepatocellular carcinoma 3; myelodysplastic syndrome 4; neoplasm malignant 3; prostate cancer 3; renal cancer 3.
Glipizide 1568: 699M, 788F	56: malignant neoplasm 7; pancreatic carcinoma 6; prostate cancer 5; colon cancer 4; myelodysplastic syndrome 4; bladder cancer 3; lung malignant neoplasm 3; breast cancer 2.
Repaglinide 2355: 1042F, 1216M	40: pancreatic carcinoma 6; pancreatic carcinoma metastatic 5; breast cancer 2; colon cancer 2; leucemia 2; lung malignant neoplasm 2; malignant neoplasm progression 2; metastasis to liver 2; myelodysplastic syndrome 1; prostate cancer 1; bladder cancer 1.
Nateglinide 614: 297F, 275M	18: malignant neoplasm 3; pancreatic carcinoma 2; acute myeloid leucemia 2; bladder cancer 2; lung malignant neoplasm 2; myelodysplastic syndrome 1.
Mitiglinide	No data
Gliclazide 3731: 1820F, 1607M	97: pancreatic carcinoma 14; malignant neoplasm 9; myelodysplastic syndrome 4; metastasis to liver 4; bladder neoplasm 2; breast cancer 2; chronic myeloid leucemia 2; hepatic cancer 2; hepatocellular carcinoma 3; lung neoplasm malignant 2; malignant neoplasm progression 2; prostate cancer 2; renal cancer 1.
Tolbutamide	No data
Zoledronic a. 42387: 28478F, 11568M	4959: breast cancer 379; neoplasm malignant 187; prostate cancer 141; lung neoplasm malignant 170; colon cancer 54; myelodysplastic syndrome 45; adenocarcinoma 44; pancreatic carcinoma 40; bladder cancer 33; renal cancer 23; hepatic cancer 26; ovarian cancer 23; cervix carcinoma 6; chronic myeloid leucemia 2; pancreatic neoplasm 3; hepatocellular carcinoma 3.
Minoxidil 6165: 3536F, 2151M	110: breast cancer 23; skin cancer 13; neoplasm malignant 11; basal cell carcinoma 5; lymphoma 5; hepatic cancer 4; meningioma 3; bladder transitional carcinoma 3; colon cancer 3; lung neoplasm malignant 3; brain 2; brain neoplasm 2; adenocortical carcinoma 1; prostate cancer 2; pancreatic carcinoma 2; benign skin neoplasm 1; myelodysplastic syndrome 1; ovarian cancer 1.
Diazoxide 445: 212F, 188M	10: insulinoma 4, liver metastasis 4, malignant neoplasm progression 2.
Nicorandil 1895: 828F, 1006M	15: gastric cancer 2, pancreatic carcinoma 1.

The sulfonylureas and glinides targeting the Kir6.2-Sur1 subunits showed an elevated case report number for pancreatic carcinoma and bladder cancers, but a low number of reports for common cancers.

The Kir6.1/2-Sur2A/B antagonist zoledronic acid showed instead higher case report numbers for breast > malignant neoplasm > prostate > lung neoplasm malignant > myelodysplastic syndrome > bladder > renal > ovarian > cancers and lower number of cancer cases for other cancer types including pancreatic carcinoma (Table 4).

**FIGURE 6:**
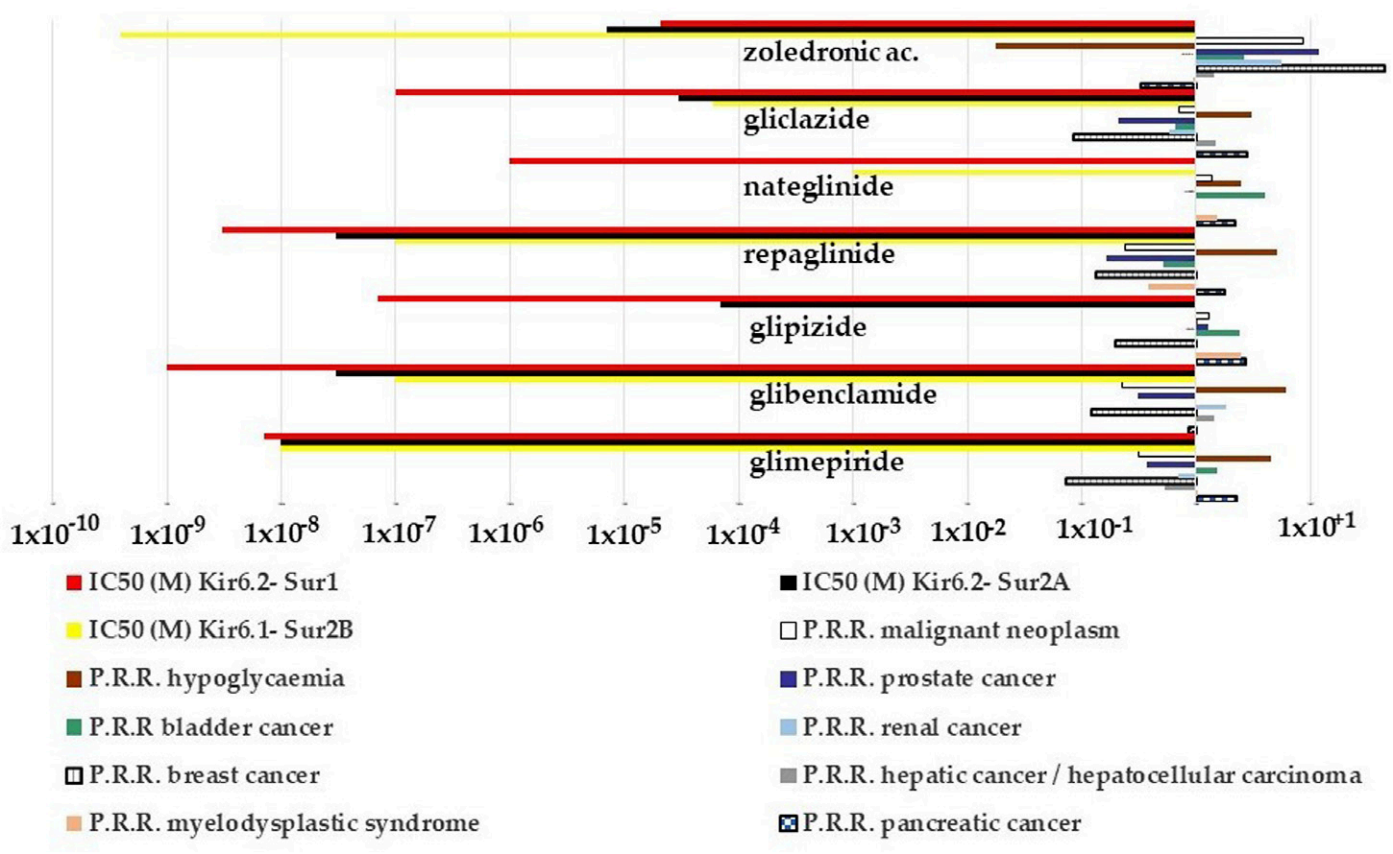
P.R.R. plot of different cancer types and hypoglycemia risks of the KATP channel blockers and their IC_50_ values to block recombinant KATP channel subunits expressed in the cell line. The P.R.R. of hypoglycemia was > 2 for the sulfonylureas and glinides but not for zoledronic acid; the P.R.R. values for the pancreatic carcinoma were > 2 for the glipizide, glimepiride, nateglinide, and gliclazide; glipizide and nateglinide had a P.R.R. >2 for bladder cancer, glipizide also for the myelodysplastic syndrome. P.R.R. values of breast and prostate cancers were <1 for all sulfonylureas and glinides. P.P.R. values of zoledronic acid > 2 for all cancer types but lower values for pancreatic, renal, and bladder cancers. It also shows a low hypoglycemia risk. P.R.R. > 1 indicates a high risk of A.D.Rs., and lower values indicate a low risk of A.D.Rs. P.R.R. values > 2 are considered statistically significant.

We, therefore, evaluated the possible relationship between the P.R.R. of the drugs for different cancer types and for hypoglycemia and their capability to block the KATP channel subunits. The P.R.R. for pancreatic cancers of glipizide, gliclazide, and nateglinide appears to be inversely correlated with their IC_50_ to block the recombinant Kir6.2-Sur1 subunits with the glipizide, gliclazide, and nateglinide showing the highest cancer risk and the lowest hypoglycemia risk (Figure 6) within the KATP channel blockers including zoledronic acid. On the opposite, repaglinide, glibenclamide, and glimepiride showed the lowest P.R.R. for pancreatic cancer and the highest hypoglycemia risk (Figure 6).

Zoledronic acid, a non-selective KATP channel blocker (Maqoud et al., 2021), showed no risk for pancreatic cancer, moderate cancer risk with P.P.R. values ≥ 2 for several cancer types including bladder and renal cancers, but high cancer risk with P.P.R. values > 10 for breast and prostate cancers (Figure 6).

Within the KATP channel openers, minoxidil showed a higher number of A.D.Rs. within the class of breast cancer, skin cancer, and malignant neoplasms while diazoxide did not (Table 4). Due to the low number of events, the P.R.R. of the drug class was calculated aggregated only for the S.O.C. (neoplasm benign, malignant, and unspecified disorders) of the KATP channel openers. The P.R.R. values were 0.42, 1.45, and 1.65 for nicorandil, diazoxide, and minoxidil, respectively. None of these drugs induced a statistically significant cancer risk within the class.

Therefore, the minoxidil-induced upregulation of the KATP channels composed of the Kir6.1/2-Sur2A/B subunits can be associated with high cancer risk in some common cancer including breast cancer in humans but not pancreatic cancer in line with the lack of expression of these subunits in pancreatic beta cells. While the sulfonylureas and the glinides blocking the Kir6.2-Sur1 subunits highly expressed in this tissue show high pancreatic cancer risk inversely related to the hypoglycemia risk. In line with this observation, the Kir6.2-Sur1 opener diazoxide shows no cancer risk including pancreatic cancer.

### Omics analysis

We, therefore, investigated the available omics data related to KATP channel genes and their involvement in cancers.

### Browsing integrated databases

The structured databases available at the NCBI report four human genes whose official name is ATP-sensitive potassium channel subunit (KATP)ʼ *ABBC8* (Sur1), *ABCC9* (Sur2 A/B), *KCNJ11* (Kir6.2), and *KCNJ8* (Kir6.1) genes. In detail, we focused our attention on data concerning mutations, literature, BioProjects, and gene expression where high-throughput data could open new scenarios regarding the role of these genes in cancer.

### BioProjects

The BioProject database collects complete and in-progress large-scale data regarding projects about genome sequencing, expression, metagenomic, annotation, and mapping of bio-data. Hence, BioProject has a central role to reach molecular and literature databases. However, searching for data correlating the gene name and the term cancer yielded data only for the *ABCC8* gene. More in one BioProject (PRJNA541505) regarding *ABCC8* is in progress, but at present, no relevant data are available. In PRJNA187483: GEO: GSE43807, BioProject aimed to investigate associations between ABC transporter expression and outcome of breast cancer patients by qPCR in post-treatment and non-neoplastic tumor samples from 68 breast cancer patients treated with neoadjuvant chemotherapy. The data produced underlined the significant associations of *ABCC1* and *ABCC8* levels in tumors with the grade and expression of hormone receptors (Hlaváč et al., 2013).

### Pathogenic mutations

As far as the Variation section available in the Gene database is concerned, we have selected variants annotated in ClinVar (Landrum et al., 2018) as single-nucleotide missense mutations and validated them as pathogenic/likely pathogenic by multiple submitters. While no missense mutations are reported in *KCNJ8*, eight, twenty-seven, and five missense mutations related to *KCNJ11*, *ABCC8*, and *ABCC9*, respectively, have been observed in phenotypes prevalently related to well-known diseases such as diabetes mellitus, hyperinsulinemic hypoglycemia, and Brugada syndrome and the recently identified C.S. (Supplementary Table S1). However, no evidence regarding cancers is reported. Hence, we have moved toward an approach of data mining in literature data.

Starting from the scientific publications available in PubMed and selected by the Gene and OMIM (Amberger and Hamosh, 2017) database curators, a mining approach based on the Entrez retrieval system, as described in “*Methods—Omics analysis*,” has highlighted that besides the well-known role of the channels in diabetes and hyperinsulinemia, differential expression and mutations affecting the four genes are also correlated with some cancer types (Supplementary Table S2).

### Gene expression browsing the HPA and Pan Cancer databases

The differential expression of genes (DEGs) may be the cause or can contribute to cancer development.

Here, we report the expression values of the four genes of our interest by browsing the HPA and Pan Cancer databases. Moreover, a detailed analysis of the four genes of our interest was performed through the Kaplan–Meyer plotter platform (https://kmplot.com/analysis/).

The section “Expression” available through the “Gene database” reports the expression of the gene as measured within the HPA project (BioProject: PRJEB4337, PMID 24309898), where RNA-seq results regarding 27 different tissues from 95 human healthy individuals were produced to estimate tissue specificity of all protein-coding genes. The expression value was quantified in the number of RPKM placed.

As shown in Table 5, the data regarding healthy tissues where the RPKM is higher than 2.0 are reported for the four genes. Brain tissue is the only tissue in healthy subjects that shows significant expression values for all four genes of interest. Tumor samples TPM from Pan Cancer expression values were also reported (Table 5).

**TABLE 5:** HPA tissues from healthy samples showing high expression values and tumor samples from Pan Cancer Expression values in the range of high and middle values estimated for the four genes.

Genes and tissues	RPKM Healthy > 2 RPKM	READS COUNT Healthy	TUMOR = fold changes >10 TPM
*ABCC8*			
brain	3.342 ± 1.732	367.126	Glioma = 18 folds
adrenal	2.531 ± 0.188	223.970	

*ABCC9*			
fat	12.399 ± 0.455	1.333.521	
liver	7.26 ± 0.618	938.543	
heart	5.884 ± 1.047	1.433.293	
endometrium	5.786 ± 0.926	657.554	
gall bladder	5.417 ± 1.45	937.214	
ovary	3.177 ± 0.403	438.988	
lung	2.745 ± 0.886	503.576	
kidney	2.524 ± 1.346	279.314	
prostate	2.5 ± 0.634	342.762	
urinary bladder	2.255 ± 0.501	254.302	
esophagus	2.125 ± 1.188	372.989	
brain	2.071 ± 0.88	237.220	

*KCNJ11*			
brain	2.501 ± 1.173	106.223	Glioblastoma multiforme = 21 folds
			Glioma = 12 folds
thyroid glands	2.27 ± 2.099	195.591	
pancreas	<2	/	Pancreatic adenocarcinoma = 21 folds
kidney	<2	/	Renal cell carcinoma = 24 folds
skin	<2	/	Melanoma = 12 folds
heart	2.162 ± 0.434	169.586	
skeletal muscle	nd	/	Sarcoma = 24 folds
liver	<2	/	Hepatocellular carcinoma = 25 folds
ovary	<2	/	Ovarian adenocarcinoma = 12 folds
prostate glands	<2	/	Prostate adenocarcinoma = 14 folds

*KCNJ8*			
brain	8.825 ± 2.434	1.183.186	Glioma = 25 folds
kidney	<2	/	Chromophobe renal cell carcinoma = 24 folds
thyroid glands	<2	/	Follicular thyroid carcinoma = 13 folds
prostate glands	<2	/	Prostate adenocarcinoma = 14 folds

The values have been filtered considering only high and middle expression data whose TPM values are higher than ten, (n d: not investigated).

### Differential expression analysis

The screening of significant DEGs in a specific disease status is of great support to locating genes whose role may support the definition of a therapeutic program. To this aim, the four genes have been analyzed through the TNMPlot web tool (Bartha and Győrffy, 2021).

**FIGURE 7:**
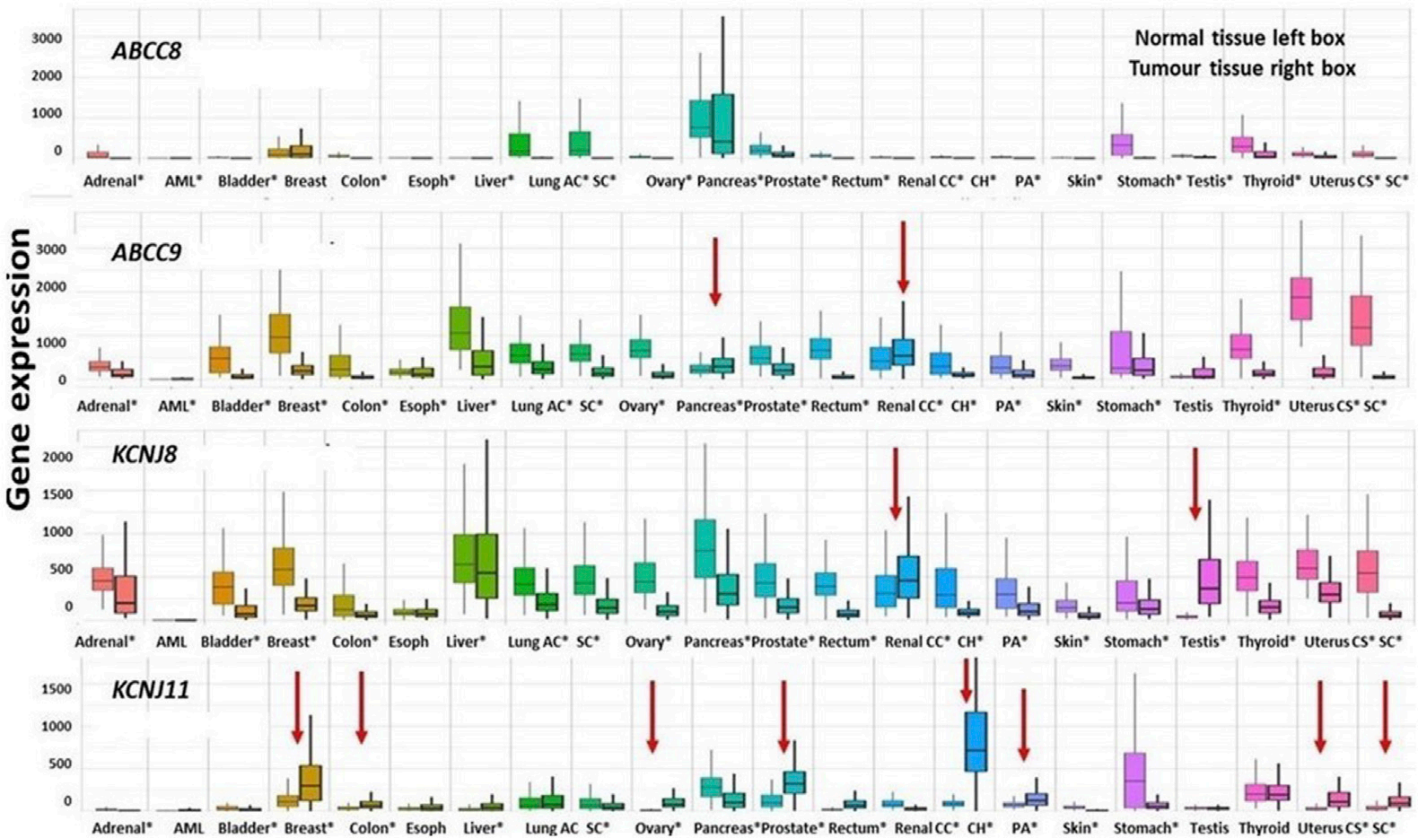
Differential expression genes are represented as boxplots of the *ABCC8*, *ABCC9*, *KCNJ8*, and *KCNJ11* genes differentially expressed in several common tumor types. From left to right, the boxplots report the differential expression of tumor tissues compared to normal tissues of the following tumors: adrenal cancer, acute myeloid leukemia (aml), bladder cancer, breast cancer, colon cancer, esophageal carcinoma, liver cancer, lung adenocarcinoma, lung squamous cell carcinoma, ovarian serous cystadenocarcinoma, pancreatic adenocarcinoma, prostate adenocarcinoma, rectum adenocarcinoma, renal clear cell carcinoma, kidney chromophobe, kidney renal papillary cell carcinoma, skin cutaneous melanoma, testicular germ cell tumors, thyroid carcinoma, uterine carcinosarcoma, and uterine corpus endometrial carcinoma. Significant differences are observed from a Mann–Whitney *U* test (*p < 0.01). The four genes were downregulated in the great part of the considered cancer types of exception made for those marked with red arrows which are upregulated.

The *ABCC8* genes show a significant downregulation vs*.* non-cancerous tissues in almost all the common cancers, *ABCC9* is also prevalently downregulated except for the kidney renal clear cell carcinoma and pancreatic cancer which showed an upregulation vs*.* the non-cancerous tissues (red arrow in Figure 7). The *KCNJ8* gene was also downregulated vs. controls except for testicular germ cell\tumors and kidney renal clear cell carcinoma that showed an upregulation of the gene expression.

The *KCNJ11* gene was markedly upregulated in kidney chromophobe and prostate adenocarcinoma, kidney renal papillary cell carcinoma, breast cancer, uterine carcinosarcoma, and uterine corpus endometrial carcinoma (Figure 7).

### Pan Cancer survival analysis

The pathogenic and clinical significance of the observed gene expression changes was evaluated. We found that the upregulation of the *ABCC8* gene was correlated with overall survival in the lung and pancreatic ductal adenocarcinoma with a low risk of progression in white male populations (H.R. values = < 1). Also, the *ABCC9* gene expression was correlated with a low risk of progression in pancreatic ductal adenocarcinoma in white females (H.R. values = < 1) (Table 6).

**TABLE 6:** Hazard ratio (H.R.) of disease progression values for genes and cancer type whose survival KmPlot analyses show significant results (*p* < 0.05).

		*ABCC8*		*ABCC9*		*KCNJ8*		*KCNJ11*	
Gender/race	Tumor type	*p*-value	H.R.	*p*-value	H.R.	*p*-value	H.R.	*p*-value	H.R.
Male/white	Lung adenocarcinoma	0.022	0.45						
	Lung squamous cell carcinoma			0.038	1.76	0.027	1.78		
	Pancreatic ductal adenocarcinoma	0.04	0.4						
	Stomach adenocarcinoma	0.02	2.34						
Male/Asian	Liver hepatocellular carcinoma	0.04	1.96	0.01	0.24			0.0005	3.5
	Stomach adenocarcinoma					0.03	4.5		
Male/black/African–American	Head–neck squamous cell carcinoma	0.004	0.09	0.003	0.2	0.005	0.3	0.01	0.3
	Kidney renal papillary cell carcinoma							0.03	8.8
	Lung adenocarcinoma							0.03	0.1
Female/white	Bladder carcinoma			0.041	2.67				
	Breast cancer			0.012	2.02				
	Lung adenocarcinoma								
	Lung squamous cell carcinoma	0.03	2.82						
	Pancreatic ductal adenocarcinoma			0.032	0.27				
	Stomach adenocarcinoma					0.045	2.74		
Female/Asian	Uterine corpus endometrial carcinoma			0.014	0				
	Liver hepatocellular carcinoma					0.03	0		
	Breast cancer							0.04	0
	Stomach adenocarcinoma							0.04	0
Female/black/African–American	Lung adenocarcinoma			0.01	0.11				
	Lung adenocarcinoma					0.02	0.09		
	Lung adenocarcinoma					0.03	0.2		
	Breast cancer							0.01	0.4
	Uterine corpus endometrial carcinoma							0.005	3.6

An opposite trend (H.R. values = > 1) of the *ABCC8* gene was observed in the stomach and lung squamous adenocarcinoma in white males and females, respectively. Similarly, the *ABCC9* and *KCNJ8* genes’ upregulation was associated with a reduced probability of survival in lung squamous cell carcinoma in males, and the *ABCC9* gene also in bladder and breast cancers in white female patients. The *KCNJ11* gene was significantly associated with negative prognosis in Asian males, black men, and black females in liver hepatocellular carcinoma, kidney renal papillary cell carcinoma, and uterine corpus endometrial carcinoma, respectively (Table 6).

We further tested for a possible association between known gene variants and cancers. The previous meta-analysis indeed shows that *KCNJ11* rs5219 is a common variant for type II diabetes (T2D) risk in the world population and has a similar effect on the susceptibility risk to T2D in the Europeans and East Asian, the Japanese (Kuruma et al., 2014). We tested for the known common variants associated with (T2D), cardiovascular diseases, and race in the general European population (rs5219), the French (rs1799859), the Turks (rs1799854 and rs1799859), and the Iranians (rs757110) that is, however, associated with a decreased T2D risk in the British. In non-Caucasian populations, the KATP variants are associated with increased T2D risk in the Japanese (rs5219 and rs757110), the Mongolians (rs1799858 and rs2074308), the Indians (chr11:17417205C > T and rs5210), and the Nigerians (rs1799854) rather than the Punjab population of India (rs1799854 and rs1801261) and the sub-Saharan Africans (rs5219) in Ghana and the Nigerians. The KATP variants are also associated with T2D-related stroke in the Poles (e.g., rs1799854), coronary atherosclerotic heart disease (CAD) in the British (rs757110 and rs61688134), heart failure (HF) in the Ukrainians (rs5210, rs5219, and rs757110) and the Americans (rs5219), and atrial fibrillation (AF) in the Americans (rs72554071) (Liu et al., 2022).

*KCNJ11* rs5219 showed no association with pancreatic cancer in the Japanese population (Kuruma et al., 2014) and a weak association with CRC cancer risk (Cheng et al., 2011). *ABCC8* rs757110 has been associated with longevity in the Korean population (Park et al., 2009).

However, we currently failed to evidence a significant association of known variants reported in the databases with cancers.

## Discussion

In the present work, immunohistochemical investigations showed for the first time an abnormally elevated reactivity of the Sur2A subunit in tumor samples derived from minoxidil-induced renal cancer in male rats and the canine female breast cancer, a spontaneous animal model of disease. A sub-chronic high-dose topical administration of the minoxidil solution (0.777–77.7 mg/kg/day) provoked renal cancer in male rats, while breast cancer was found in female dogs admitted to the hospital for diagnosis. Minoxidil treatment is a pharmacological animal model of C.S. This disorder is associated with G.O.F. mutations of the *ABCC9* and *KCNJ8* genes, and some cases of familial pituitary adenoma were found in C.S. families (Marques et al., 2018a; Marques et al., 2018b).

In either animal’s models of cancer, the cytosolic compartment of the renal cells was markedly stained with Sur2A-mAb, while the other subunits were normally distributed and not upregulated in different cell compartments and renal districts. Other than the localization at the surface membrane, the minoxidil-sensitive Kir6.2-Sur2A/B complex has been found expressed in several cell compartments including the sarcoplasmic reticulum (Shorter et al., 2008), endoplasmic reticulum (Salari et al., 2015), mitochondria (Garlid and Halestrap, 2012), and endosome/lysosomes (Bao et al., 2011). To date, the role of the Sur2B variant can be considered. Sur2 is located in proximal tubular epithelial cells, and Sur2B is also widely expressed in the kidney, particularly in the proximal tubule, ascending limb, and the collecting duct, where it presumably mediates, in part, K+ transport and sulfonylureas induce apoptosis in the proximal tubular epithelial cells (Chutkow et al., 2002; Szamosfalvi et al., 2002; Zhou et al., 2008; Zhang et al., 2018). Spontaneous kidney tumors are slow growing and are found sporadically in untreated rodents, mostly in elderly subjects, and have an incidence of about 0.1% (Giknis and Clifford, 2013). They are more evident in animals in which high doses of genotoxic or carcinogenic chemicals have been administered causing degenerative and proliferative and neoplastic renal tubular alterations (Hall et al., 2007). Renal tumors have histological characteristics similar to those of humans and are classified into adenomas and carcinomas of the renal tubules, transitional or squamous carcinomas deriving from the urothelium of the renal pelvis, renal tumors originating from the mesenchymal and adipose cells (lipoma and liposarcoma) originating in the interstitial connective tissue and tumors of embryonic origin, and nephroblastoma (Hall et al., 2007). It should be noted that in our work, minoxidil treatment at therapeutic doses in the rat did not cause renal cancer reactions. Furthermore, renal cancer is a rare condition in humans in comparison to breast or ovarian cancers limiting the observation of the relative number of renal A.D.Rs. reported in the database.

Also, an elevated immunohistochemical reactivity of the tumor cells to the Sur2A-mAb was found in the cytosolic compartment of the breast cancer cells suggesting elevated protein activity in the G3 cells but not in the G2 or G1 cells in female canine breast cancer biopsies. The *ABCC9* gene is associated with poor prognosis in women with breast cancer, and the minoxidil treatment is associated with an elevated number of breast cancer cases that we reported here. Minoxidil is a selective KATP channel opener targeting Kir6.1/2-Sur2A-B with no action on the Kir6.2-Sur1 subunits, while diazoxide, a KATP channel opener, targeting the pancreatic Kir6.2-Sur1 subunits does not show any breast cancer reactions. Minoxidil showed a P.R.R. for cancer of 1.65 within the KATP channel openers with only one case of ovarian cancer in line with the proposed role of the *ABCC9*/Sur2 as a positive prognostic factor in this type of cancer (Fukushiro-Lopes et al., 2020).

In addition, omics data showed that the *ABCC9*, *KCNJ11*, and *KCNJ8* genes were indeed upregulated in the kidney renal clear cell carcinoma and the *KCNJ11* gene in kidney chromophobe. The *ABCC9* and *KCNJ8* genes’ upregulation was associated with a reduced probability of survival in lung squamous cell carcinoma in males and the *ABCC9* gene also in bladder and breast cancers in white female patients. The *KCNJ11* gene was significantly associated with a negative prognosis in Asian males, black men, and black females in liver hepatocellular carcinoma, kidney renal papillary cell carcinoma, and uterine corpus endometrial carcinoma (Table 6).

Pharmacology/pharmacovigilance investigation showed that the KATP channel blockers sulfonylureas and glinides with high affinity and selective for Kir6.2-Sur1 channels within the drug class had the highest P.R.R. values for pancreatic cancers in unselected and uncontrolled population. Accordingly, diazoxide, a KATP opener targeting the Kir6.2-Sur1 of pancreatic beta cells, does not show cancer reactions including pancreatic cancer. These findings are in line with the omics and literature data on the role of *ABCC8/*Sur1 that was found downregulated in pancreatic cancer (Mohelnikova-Duchonova et al., 2013), where the low expression of *ABCC8* is associated with poor prognosis with drug–disease interaction. This interaction may also occur under those conditions such as triple-negative breast cancer (Hlaváč et al., 2013) and lung adenocarcinoma (Wang et al., 2020), where the low expression of *ABCC8*/Sur1 has been also associated with poor prognosis. Sulfonylureas and glinides showed, in contrast, low cancer risks for breast, prostate, renal, and hepatic cancers. Transcriptomic data support the safe use of these drugs under these conditions as well as in gastric cancer or glioma, where the upregulation of the *ABCC8* gene has been associated with poor prognosis (Mao et al., 2019).

In line with these findings, zoledronic acid is a potent Kir6.1/2-Sur2A/B blocker targeting either the Kirs or Sur2 subunits, and a low-affinity blocker of the pancreatic Kir6.2-Sur1 channel (Maqoud et al., 2021; Scala et al., 2021) showed low hypoglycemia risk and low risk for pancreatic cancer within the KATP channel blockers. The elevated breast cancer risk observed with zoledronic acid is explained by the fact that this drug irreversibly blocks also the Kir6.1*-*Sur1 subunits at higher concentrations (Maqoud et al., 2021). However, in this case, the pharmacological block of the *ABCC8*/Sur1 subunit by this drug may have a deleterious action indeed, and the downregulation of the *ABCC8*/Sur1 gene has a negative prognostic role in cancers (Hlaváč et al., 2013). It should be of note that zoledronic acid also binds to additional sites in cancers, and it is a well-known potent inhibitor of human farnesyl pyrophosphate synthase, a key enzyme in the mevalonate pathway with apoptotic cell death.

Finally, the upregulation of *ABCC9*, *ABCC8*, and *KCNJ8* genes was associated with a low probability of survival with a higher risk > 2 in most of the tumors in white female populations suggesting gender-specific effects as reported in cardiovascular apparatus (Ranki et al., 2002; Brown et al., 2005) and neurons (Niu et al., 2011), where the estrogen may differently upregulate Kir6.2 and Sur2A-B in some tissues, for instance, cardiomyocyte, and downregulate KATP channel subunits in neurons from female rats with opposite effects. Estrogen- related elements are not present in the gene sequences of the KATP channel subunits suggesting a regulatory mechanism playing a role in these diseases. The gender-specific effects need to be investigated in a controlled study.

## Conclusion

The Sur2A/B subunits and their accessory subunits Kir6.1/2 have a role in renal and breast cancers as observed in our animal models. The minoxidil-induced overactivation of Kir6.1/2-Sur2A/B subunits in rats provoked a cancer reaction similar to what was observed in some cases of C.S. Omics data reporting the upregulation of the *ABCC9*, *KCNJ11*, and *KCNJ8* genes in cancers support these conclusions. The *ABCC9/*Sur2 gene is expressed in Ki67-positive cells and can be the target of drugs of therapeutic interest such as antidiabetic, cardiovascular, and anti-cancer drugs interfering with cell proliferation with drug–disease interactions. Sur2A is abnormally located in the cytosolic compartment in breast and renal animal tumor sections. We need to establish the role of the *ABCC9*/Sur2 in human renal cancer and the pathogenic coupling with oncogenes and inflammatory signaling.

Sulfonylureas and glinides showed low cancer risks for breast, prostate, renal, and hepatic cancers and can be proposed in gastric cancer or in glioma, where the upregulation of the *ABCC8* gene has been associated with poor prognosis (Mao et al., 2019). However, these drugs may be not recommended in diabetic patients affected by pancreatic cancer (Mohelnikova-Duchonova et al., 2013), triple-negative breast cancer (Hlaváč et al., 2013), and lung adenocarcinoma (Wang et al., 2020), where the low expression of *ABCC8*/Sur1 is associated with poor prognosis with drug–disease interaction. Glibenclamide, repaglinide, and glimepiride showed instead the lowest cancer risk within the KATP channel blockers, and they can be more safely used in type II diabetic patients affected by cancers. The fact that the lower cancer risk of these drugs is associated with the hypoglycemia risk can be explained by the fact that hypoglycemia is *per se* antiproliferative. Glibenclamide reverts the cardiovascular phenotype in C.S., and it can be safely proposed in these patients affected also by cancers.

It should be of note that type II diabetic patient populations show *per se* an elevated cancer risk vs. the general, non-diabetic population, and the drug treatment with KATP channel blockers is correlated with this risk.

Our data also support the safe use of zoledronic acid as a KATP channel blocker (Maqoud et al., 2021) in the treatment of macroadenoma in C.S. associated with G.O.F. mutations of the *ABCC9* and *KCNJ8* genes or in cancers in which these subunits were associated with poor prognosis. However, the use of this drug may not be recommended in epithelial ovarian cancer and relative metastatic spread, where the *ABCC9* upregulation was significantly associated with longer progression-free survival of female patients (Fukushiro-Lopes et al., 2020) or in renal and breast cancers showing downregulation of the gene with drug–disease interaction.

However, given that results indicative of correlations between the genes here of interest and the stages of the onset of tumor states, it is considered appropriate to investigate this aspect further through the design and implementation of *ad hoc* genomic studies based on data mining and statistical estimation approaches. Cancer signals emerged in this work associated with the KATP channel subunit genes that need to be experimentally evaluated in human/animal biopsies.

## Data Availability

The datasets presented in this study can be found in online repositories. The names of the repository/repositories and accession number(s) can be found in the article/Supplementary Material.
