# Supplementary material for: Immunohistochemical, pharmacovigilance, and omics analyses reveal the involvement of ATP-sensitive K+ channel subunits in cancers: role in drug–disease interactions
Source: Front Pharmacol. 2023 Apr 25;14:1115543. doi: 10.3389/fphar.2023.1115543 (PMC10167295; doi:10.3389/fphar.2023.1115543)
Supplement: Supplementary file 1 [file Table1.DOCX]

Supplementary Material

Immunohistochemical, pharmacovigilance and omics analyses reveal the involvement of ATP-sensitive K^+^ channel subunits in cancers: role in drug-disease interactions

Fatima Maqoud^1,†^, Nicola Zizzo^2,†^, Marcella Attimonelli^3^, Antonella Tinelli^2^, Giuseppe Passantino^2^, Marina Antonacci^1^, Girolamo Ranieri^4,^*, and Domenico Tricarico.

*** Correspondence:** Dr. Girolamo Ranieri email: [giroran@tiscali.it](mailto:giroran@tiscali.it); Prof. Domenico Tricarico, email: [domenico.tricarico@uniba.it](mailto:domenico.tricarico@uniba.it)

**Table 1S.** Missense Pathogenic not Cancer Variants (mutations) annotated in ClinVar and validated as pathogenic by multiple submitters

| **Gene** | **Variant genomic location** | | **Variant transcript location** | **Protein change** | **Condition(s)** |
| --- | --- | --- | --- | --- | --- |
| *KCNJ11* | NC_000011.10:17387247:C:T | NM_000525.4(KCNJ11):c.844G>A (p.Glu282Lys) | | E282K, E195K | Maturity-onset diabetes of the young, type; Hyperinsulinemic hypoglycemia, familial; Type II diabetes mellitus; Permanent neonatal diabetes mellitus; Transient neonatal diabetes mellitus; Hyperinsulinemic hypoglycemia, familial |
| *KCNJ11* | NC_000011.10:17387315:T:C | NM_000525.4(KCNJ11):c.776A>G (p.His259Arg) | | H259R, H172R | Hyperinsulinemic hypoglycemia, familial |
| *KCNJ11* | NC_000011.10:17387490:G:A | NM_000525.4(KCNJ11):c.601C>T (p.Arg201Cys) | | R201C, R114C | Diabetes mellitus\|Permanent neonatal diabetes mellitus; Permanent neonatal diabetes mellitus; Transient neonatal diabetes mellitus |
| *KCNJ11* | NC_000011.10:17387916:C:T | NM_000525.4(*KCNJ11*):c.175G>A (p.Val59Met) | | V59M | Permanent neonatal diabetes mellitus; Permanent neonatal diabetes mellitus; Neonatal insulin-dependent diabetes mellitus |
| *KCNJ11* | NC_000011.10:17387210:G:A | NM_000525.4(*KCNJ11*):c.881C>T (p.Thr294Met) | | T294M, T207M | Transient neonatal diabetes mellitus; Hyperinsulinemic hypoglycemia, familial; Hyperinsulinemic hypoglycemia, familial; Maturity-onset diabetes of the young, type 13\|Transient neonatal diabetes mellitus; Type II diabetes mellitus; Permanent neonatal diabetes mellitus |
| *KCNJ11* | NC_000011.10:17387942:C:T | NM_000525.4(*KCNJ11*):c.149G>A (p.Arg50Gln) | | R50Q | Neonatal diabetes mellitus |
| *KCNJ11* | NC_000011.10:17387412:C:T | NM_000525.4(*KCNJ11*):c.679G>A (p.Glu227Lys) | | E227K, E140K | Maturity-onset diabetes of the young, type 13\|\|Diabetes mellitus\|Transient neonatal diabetes mellitus 3 |
| *KCNJ11* | NC_000011.10:17387489:C:T | NM_000525.4(*KCNJ11*):c.602G>A (p.Arg201His) | | R201H, R114H | Permanent neonatal diabetes mellitus\|Permanent  neonatal diabetes mellitus 2\|\|Neonatal insulin-dependent diabetes mellitus\|Transient neonatal diabetes mellitus 3 |
| *ABCC8* | NC_000011.10:17395619:C:T | NM_000352.6(*ABCC8*):c.4297G>A (p.Gly1433Ser) | | G1432S, G1433S, G1434S, G1455S |  |
| *ABCC8* | NC_000011.10:17395213:C:T | NM_000352.6(*ABCC8*):c.4369G>A (p.Ala1457Thr) | | A1456T, A1457T, A1458T, A1479T | Hyperinsulinemic hypoglycemia, familial, 1 |
| *ABCC8* | NC_000011.10:17463456:A:T | NM_000352.6(*ABCC8*):c.560T>A (p.Val187Asp) | | V187D | \|Hyperinsulinemic hypoglycemia, familial, 1 |
| *ABCC8* | NC_000011.10:17393123:C:T | NM_000352.6(*ABCC8*):c.4613G>A (p.Arg1538Gln) | | R1538Q, R1539Q, R1560Q, R1537Q |  |
| *ABCC8* | NC_000011.10:17395913:C:A | NM_000352.6(*ABCC8*):c.4136G>T (p.Arg1379Leu) | | R1379L, R1380L, R1378L, R1401L | Neonatal diabetes mellitus |
| *ABCC8* | NC_000011.10:17442773:G:A | NM_000352.6(*ABCC8*):c.1576C>T (p.Arg526Cys) | | R526C, R525C | Hyperinsulinemic hypoglycemia, familial, 1 |
| *ABCC8* | NC_000011.10:17395663:C:T | NM_000352.6(*ABCC8*):c.4253G>A (p.Arg1418His) | | R1418H, R1419H, R1417H, R1440H | Hyperinsulinemic hypoglycemia, familial, 1\| |
| *ABCC8* | NC_000011.10:17395892:G:A | NM_000352.6(*ABCC8*):c.4157C>T (p.Ser1386Phe) | | S1386F, S1387F, S1385F, S1408F |  |
| *ABCC8* | NC_000011.10:17442848:C:T | NM_000352.6(*ABCC8*):c.1501G>A (p.Glu501Lys) | | E501K, E500K | Hyperinsulinemic hypoglycemia, familial, 1 |
| *ABCC8* | NC_000011.10:17393108:A:G | NM_000352.6(*ABCC8*):c.4628T>C (p.Leu1543Pro) | | L1543P, L1544P, L1565P, L1542P | Hereditary hyperinsulinism\| Hyperinsulinemic hypoglycemia, familial, 1 |
| *ABCC8* | NC_000011.10:17395914:G:A | NM_000352.6(*ABCC8*):c.4135C>T (p.Arg1379Cys) | | R1379C, R1380C, R1378C, R1401C | Transient neonatal diabetes mellitus 2, Monogenic diabetes Type II diabetes mellitus Permanent neonatal diabetes mellitus |
| *ABCC8* | NC_000011.10:17476714:A:T | NM_000352.6(*ABCC8*):c.62T>A (p.Val21Asp) | | V21D | Familial hyperinsulinism\|Hereditary  hyperinsulinism\|Hyperinsulinemic hypoglycemia, familial, 1\| |
| *ABCC8* | NC_000011.10:17394332:C:T | NM_000352.6(*ABCC8*):c.4478G>A (p.Arg1493Gln) | | R1493Q, R1494Q, R1492Q, R1515Q | Hyperinsulinemic hypoglycemia, familial, 1\| |
| *ABCC8* | NC_000011.10:17461721:C:T | NM_000352.6(*ABCC8*):c.683G>A (p.Gly228Asp) | | G228D | \|Hyperinsulinemic hypoglycemia, familial, 1\|Familial hyperinsulinism |
| *ABCC8* | NC_000011.10:17474955:G:A | NM_000352.6(*ABCC8*):c.220C>T (p.Arg74Trp) | | R74W | \|Hereditary  hyperinsulinism\|Hyperinsulinemic hypoglycemia, familial, 1 |
| *ABCC8* | NC_000011.10:17404523:C:T | NM_000352.6(*ABCC8*):c.3545G>A (p.Arg1182Gln) | | R1182Q, R1183Q, R1181Q, R1204Q | \|Transient neonatal diabetes mellitus 2\|Neonatal diabetes mellitus |
| *ABCC8* | NC_000011.10:17470181:C:T | NM_000352.6(*ABCC8*):c.331G>A (p.Gly111Arg) | | G111R | Familial  hyperinsulinism\|\|Hyperinsulinemic hypoglycemia, familial, 1 |
| *ABCC8* | NC_000011.10:17394333:G:A | NM_000352.6(*ABCC8*):c.4477C>T (p.Arg1493Trp) | | R1494W, R1493W, R1515W, R1492W | \|Hereditary hyperinsulinism\|Hyperinsulinemic hypoglycemia, familial, 1 |
| *ABCC8* | NC_000011.10:17395658:G:A | NM_000352.6(*ABCC8*):c.4258C>T (p.Arg1420Cys) | | R1421C, R1420C, R1419C, R1442C | \|Hereditary hyperinsulinism\|Hyperinsulinemic hypoglycemia, familial, 1 |
| *ABCC8* | NC_000011.10:17402669:C:T | NM_000352.6(*ABCC8*):c.3641G>A (p.Arg1214Gln) | | R1214Q, R1215Q, R1213Q, R1236Q | Familial hyperinsulinism\|Hereditary  hyperinsulinism\|\|Hyperinsulinemic hypoglycemia, familial, 1 |
| *ABCC8* | NC_000011.10:17395609:C:T | NM_000352.6(ABCC8):c.4307G>A (p.Arg1436Gln) | | R1436Q, R1437Q, R1435Q, R1458Q | Familial hyperinsulinism\|Hereditary  hyperinsulinism\|Hyperinsulinemic hypoglycemia, familial, 1\| |
| *ABCC8* | NC_000011.10:17474954:C:T | NM_000352.6(*ABCC8*):c.221G>A (p.Arg74Gln) | | R74Q | Familial hyperinsulinism\|Hyperinsulinemic hypoglycemia, familial, 1\|\|Permanent neonatal diabetes mellitus\|Type II  diabetes mellitus\|Leucine-induced hypoglycemia\|Hyperinsulinemic hypoglycemia, familial, 1\|Transient neonatal diabetes mellitus 2 |
| *ABCC8* | NC_000011.10:17463453:T:C | NM_000352.6(*ABCC8*):c.563A>G (p.Asn188Ser) | | N188S | Hereditary hyperinsulinism\|\|Hyperinsulinemic hypoglycemia, familial, 1 |
| *ABCC8* | NC_000011.10:17395851:C:T | NM_000352.6(*ABCC8*):c.4198G>A (p.Gly1400Arg) | | G1400R, G1401R, G1399R, G1422R | \|Familial hyperinsulinism\|Hyperinsulinemic hypoglycemia, familial, 1\|Hereditary hyperinsulinism\|Permanent neonatal diabetes mellitus 3 |
| *ABCC8* | NC_000011.10:17394294:C:T | NM_000352.6(*ABCC8*):c.4516G>A (p.Glu1506Lys) | | E1506K, E1507K, E1505K, E1528K | \|Hyperinsulinemic hypoglycemia, familial, 1 |
| *ABCC8* | NC_000011.10:17394378:C:T | NM_000352.6(*ABCC8*):c.4432G>A (p.Gly1478Arg) | | G1478R, G1479R, G1477R, G1500R | Hyperinsulinemic hypoglycemia, familial, 1\|\|Transient neonatal diabetes mellitus 2\|Type II diabetes mellitus\|Leucine-induced hypoglycemia\|Permanent neonatal diabetes mellitus\|Hyperinsulinemic hypoglycemia, familial, 1 |
| *ABCC8* | NC_000011.10:17402670:G:A | NM_000352.6(*ABCC8*):c.3640C>T (p.Arg1214Trp) | | R1214W, R1215W, R1213W, R1236W | Familial hyperinsulinism\|Hereditary  hyperinsulinism\|Hyperinsulinemic hypoglycemia, familial, 1\| |
| *ABCC9* | NC_000012.12:21842439:C:T | NM_020297.4(*ABCC9*):c.3347G>A (p.Arg1116His) | | R1116H, R827H | Dilated cardiomyopathy 1O\|\|Hypertrichotic  osteochondrodysplasia Cantu type |
| *ABCC9* | NC_000012.12:21842440:G:A | NM_020297.4(*ABCC9*):c.3346C>T (p.Arg1116Cys) | | R1116C, R827C | Dilated cardiomyopathy 1O\|Kleefstra syndrome 1\|\|Hypertrichotic osteochondrodysplasia Cantu type |
| *ABCC9* | NC_000012.12:21842325:C:T | NM_020297.4(*ABCC9*):c.3461G>A (p.Arg1154Gln) | | R1154Q, R865Q | Dilated cardiomyopathy 1O\|Hypertrichotic  osteochondrodysplasia Cantu type\| |
| *ABCC9* | NC_000012.12:21842326:G:A | NM_020297.4(*ABCC9*):c.3460C>T (p.Arg1154Trp) | | R1154W, R865W | \|Hypertrichotic osteochondrodysplasia Cantu  type\|Dilated cardiomyopathy 1O\|Tapered finger\|Coarse facial features\|Joint  hypermobility\|Abnormal facial shape\|Patent ductus arteriosus\|Bulbous  nose\|Micrognathia\|Depressed nasal-bridge\|Macrocephalus\|Left ventricular hypertrophy\|Large hands\|Abnormality of the face\|Low anterior hairline\|Thick upper lip vermilion\|Epicanthus |

**Table 2S.** - Data for genes *ABCC8*/Sur1, *ABCC9*/Sur2, *KCNJ8*/Kir6.1, and *KCNJ11*/Kir6.2 was retrieved by browsing scientific literature extracted from PubMed through Gene and OMIM databases.

| **Differential expression/ Mutation** | **Cancers type** | **Information on analyzed samples** | **Consequences** | **PubMed code** |
| --- | --- | --- | --- | --- |
| Up-regulation of *ABCC8* | Glioma | The information is based on the analysis of 1893 human glioma samples from four independent databases | High *ABCC8* mRNA expression can be used to predict glioma chemosensitivity, while low *ABCC8* mRNA expression can be used to predict glioma sensitivity to radiotherapy. | (Zhou et al., 2020) |
| Down-regulation of *ABCC8* | Pancreatic ductal adenocarcinoma (PDAC) | The information comes from the analysis of tumor tissues and adjacent non-neoplastic tissues obtained from 32 patients with PDAC. | Possible role of stem cells in the development and progression of PDAC. | (Mohelnikova-Duchonova et al., 2013) |
| Down-regulation of *ABCC8* | Lung adenocarcinoma (LUAD) | The information is derived from the analysis of data downloaded from the TCGA database (https://www.cancer.gov/about-nci/organization/ccg/research/structural-genomics/tcga), which contains 535 LUAD and 59 paracancerous samples. | Patients with high *ABCC8* expressions had a better prognosis compared to patients with low *ABCC8* expressions. | (Wang et al., 2020) |
| Down-regulation of *ABCC8* and *ABCC9* | Breast cancer | The information was determined in post-treatment tumor and non-neoplastic tissue samples from 68 breast cancer patients. | Down-regulation of *ABCC8* and *ABCC9* present potential modifiers of the progression and response to chemotherapy of breast cancer. | (Hlaváč et al., 2013) |
| Up-regulation of *ABCC8* | Brain tumors | The information comes from the analysis of human tissue samples from 6 glioblastoma, 12 brain metastases, 11 medulloblastoma, 9 supratentorial ependymomas, and 8 posterior fossa ependymomas | Sur1 is a potential therapeutic target for reducing neuroinflammation in adult and pediatric brain tumors. Inhibition of Sur1 induces neuronal stabilization in glioblastoma, brain metastases and posterior fossa ependymoma, and edema reduction in medulloblastoma. | **(Ocampo-Garza et al., 2019)** |
| Down-regulation of *ABCC8*, no change of *ABCC9* | Colorectal cancer (CRC) | The information comes from the analysis of 51 samples of primary tumor of human colorectal carcinoma and paired distant unaffected mucosa collected from CRC patients diagnosed | The differential level of *ABCC8* gene expression can be considered a tool for assessing tumor aggressiveness and as a predictor of the outcome of chemotherapy. | (Hlavata et al., 2012) |
| Up-regulation of *KCNJ11* and *ABCC9* | Cervical cancer | Human cervical cancer biopsies, n.74 | A potential association between increased Kir6.2 / Sur2 expression and invasion of the lymphovascular space. The ATP K-channel blocker glibenclamide reduced the proliferation of cervical cancer cell lines. The ATP K-channel subunits (Kir6.2 and Sur2) could potentially represent tools for the diagnosis and treatment of cervical cancer. | (Vázquez-Sánchez et al., 2018) |
| Up-regulation of *KCNJ8* and *ABCC8* | Glioma | 20 human glioma biopsies | The Kir6.2 and Sur1 subunits of the K ATP channel are involved in the proliferation of U87 and U251 glioma cells. The K ATP channel inhibitors significantly reduced the growth curve. On the other hand, K ATP channel agonists promoted the proliferation of U87 and U251 cells. | (Huang et al., 2009) |
| Down-regulation of *ABCC9* | Triple-negative breast cancer (TNBC) | 103 TNBC samples (seven of which with corresponding paracancer samples) | | (Zhang et al., 2020) |
| Up-regulation of *ABCC8* and *ABCC9* | Gastric cancer (GC) | Analysis of a transcriptomic dataset of 1065 CG patients collected in 3 major medical centers in Berlin, Bethesda, and Melbourne. | The high expression of most ABCC family members including *ABCC8/9* suggested a poor prognosis of GC*. ABCC8, ABCC9* along with other *ABCCs (ABCC1, ABCC3, ABCC7,* and *ABCC10*) may be potential biomarkers of prognosis for GC | (Mao et al., 2019) |
| Down-regulation of *ABCC9* | Prostate cancer(PCa) | Analysis of prostate tissue samples from 104 PSA-screened at the Vilnius University Urology Center from 2008 to 2014. | Changes in ABC gene expression including *ABCC9* may favor the development of a progressive PCa phenotype resistant to antiandrogen therapy. | (Demidenko et al., 2015) |
| Up-regulation of *KCNJ8* | Adenocarcinoma of the esophagus (EAC) | Samples from 40 patients | Differential expression can be considered as the signature of the marker and can contribute to the early detection of cancer cells, to develop better detection of esophageal cancer concepts for earlier therapy and a more favorable prognosis. | (Warnecke-Eberz et al., 2016) |
| Frameshift mutation (c.3512delT, p.L1171fs) in the *ABCC8* gene located on chromosome 11p15.1 | Hepatobla  stoma | Case report: a child with congenital focal hyperinsulinism with a paternal hereditary *ABCC8* mutation and paternal uniparental mosaic 11p disomy | | (Calton et al., 2013) |
| Genetic alterations in noncoding regions of *ABCC8* and the coding regions (synonymous, missense amino acid changes, and frameshift mutations) | Breast cancer | Blood DNA samples from 24 breast cancer patients | Exome sequencing identified several functionally relevant alterations in *ABCC8* genes | (Soucek et al., 2015) |
| Missense amino acid changes mutation in the *ABCC9* | Non-functioning pituitary adenomas and Cantú syndrome | Case report of a five-member three-generation family with Cantú syndrome due to a novel missense variant in *ABCC9* gene | Presence of endocrinopathy without any abnormality in the growth hormone (GH) axis | (Marques et al., 2018b) |
| Somatic mutations in *ABCC9* (6%) | Endometrial cancer | Tumor tissue samples of 53 serous cases, 23 clear cell cases, 67 endometrioid cases, and 18 cases of mixed histology were obtained mostly from the Cooperative Human Tissue Network, a small part of the Biosample Repository at the Fox Chase Cancer Center, or from Oncomatrix, Inc (San Marcos, CA) | | (Le Gallo et al., 2012) |
| Somatic mutations in *ABCC9* | Gastric cancer (GC) | The analysis is based on the clinical information (age, sex, histological grade, survival status, and stage) and RNA sequencing (RNA-seq) of n = 407 with GC that was retrieved from the Cancer Genome Atlas (TCGA ) | *ABCC9* tumor mutational burden (TMB) is associated with GC prognosis. | (Zhang et al., 2022) |
| Mutations in *ABCC9* | Large granular lymphocyte leukemia (LGL) | Analysis of paired whole-exome and transcriptome sequencing information in the largest LGL cohort to date, which included 105 patients | | (Cheon et al., 2022) |
| Mutations in *ABCC9* | Breast cancer | The analysis is based on the DNA methylation data downloaded from the TCGA Assembler 2 based on the race-specific metadata of the TCGA - Breast Invasive Carcinoma (TCGA-BRCA) project from the Genomic Data Commons (GDC) data portal. | Mutations in the *ABCC9* gene, *SHISA3,* and *POU4F1* are considered potential stage-specific detection biomarkers of breast cancer. | (Ivan et al., 2021) |
| Mutations in *ABCC8* | Pancreatic neuroendocrine tumors (PanNETs) | Analysis of the data collected from the ICGC 1 database and the NCBI GEO database based on 104 samples of Italian and Austrian patients. | Mutations in the *ABCC8* gene along with four other genes are related to the development and prognosis of PanNETs and can serve as specific biomarkers and therapeutic targets. | (Xiao et al., 2019) |
| Up-regulation of *KCNJ11* | Hepatocel-lular carcinoma (HCC) | Data obtained from the study of tumor tissue samples from the Rizhao City People's Hospital were collected between 2012 and 2015, in addition to data extracted from the TCGA. | Greater expression of *KCNJ11* predicts a poor prognosis in HCC | (Zhang et al., 2018) |
| Up-regulation *ABCC9* | Epithelial ovarian cancer (EOC) | Study of tissue samples from 63 ovarian cancer patients diagnosed in the Pilsen University Hospital in the Czech Republic in the period 2013-2016. As a control set, 14 ovarian tissue samples without tumor cells were used from Motol University Hospital in Prague, Czech Republic. | High expressions of *ABCC9* and other genes were significantly associated with longer progression-free survival of patients; Furthermore, In intraperitoneal metastases, expression of all of these genes was highly correlated and indicated a prognostic profile. Differential expression of the *ABCC9* gene can be proposed as a novel marker of ovarian cancer progression and metastatic spread and can potentially be useful as a therapeutic target. | (Elsnerova et al., 2017) |
